# Insights into Interlayer Dislocation Augmented Zinc‐Ion Storage Kinetics in MoS_2_ Nanosheets for Rocking‐Chair Zinc‐Ion Batteries with Ultralong Cycle‐Life

**DOI:** 10.1002/smll.202410408

**Published:** 2025-01-09

**Authors:** Muruganandham Hariram, Pankaj K. Pal, Anusree S. Chandran, Manikantan R. Nair, Manoj Kumar, Mukhesh K. Ganesha, Ashutosh K. Singh, Basundhara Dasgupta, Saurav Goel, Tribeni Roy, Prashanth W. Menezes, Debasish Sarkar

**Affiliations:** ^1^ Department of Physics Malaviya National Institute of Technology Jaipur Rajasthan 302017 India; ^2^ Department of Mechanical Engineering Birla Institute of Technology and Science, Pilani (BITS Pilani) Rajasthan 333031 India; ^3^ Centre for Nano and Soft Matter Sciences Bengaluru 562162 India; ^4^ Department of Chemistry Technical University of Berlin Straße des 17 Juni 135. Sekr. C2 10623 Berlin Germany; ^5^ School of Engineering London South Bank University London SE1 0 AA UK; ^6^ University of Petroleum and Energy Studies Dehradun 248007 India; ^7^ Material Chemistry Group for Thin Film Catalysis – CatLab Helmholtz‐Zentrum Berlin für Materialien und Energie Albert‐Einstein‐Str. 15 12489 Berlin Germany; ^8^ Centre for Future Materials (CFM) University of Southern Queensland Queensland QLD 4350 Australia

**Keywords:** carbon‐dots, interlayer dislocation, interlayer expansion, MoS_2_, rocking‐chair Zinc‐ion batteries

## Abstract

Increasing attention to sustainability and cost‐effectiveness in energy storage sector has catalyzed the rise of rechargeable Zinc‐ion batteries (ZIBs). However, finding replacement for limited cycle‐life Zn‐anode is a major challenge. Molybdenum disulfide (MoS_2_), an insertion‐type 2D layered material, has shown promising characteristics as a ZIB anode. Nevertheless, its high Zn‐ion diffusion barrier because of limited interlayer spacing substantiates the need for interlayer modifications. Here, N‐doped carbon quantum dots (N‐CQDs) are used to modify the interlayers of MoS_2_, resulting in increased interlayer spacing (0.8 nm) and rich interlayer dislocations. MoS_2_@N‐CQDs attain a high specific capacity (258 mAh g^−1^ at 0.1 A g^−1^), good cycle life (94.5% after 2000 cycles), and an ultrahigh diffusion coefficient (10^−6^ to 10^−8^ cm^2^ s^−1^), much better than pristine MoS_2_. Ex situ Raman studies at charge/discharge states reveal that the N‐CQDs‐induced interlayer expansion and dislocations can reversibly accommodate the volume strain created by Zn‐ion diffusion within MoS_2_ layers. Atomistic insight into the interlayer dislocation‐induced Zn‐ion storage of MoS_2_ is unveiled by molecular dynamic simulations. Finally, rocking‐chair ZIB with MoS_2_@N‐CQDs anode and a Zn_x_MnO_2_ cathode is realized, which achieved a maximum energy density of 120.3 Wh kg^−1^ and excellent cyclic stability with 97% retention after 15 000 cycles.

## Graphical abstract

MoS_2_ nanosheets enriched with interlayer dislocations via N‐CQDs incorporation deliver high specific capacity (258 mAh g^−1^ at 0.1 A g^−1^) and outstanding cycle stability (94.5% retention after 2000 cycles) in Zn‐ion batteries. Molecular dynamics and ex situ analyses reveal mechanistic insights, with MoS₂@N‐CQDs achieving an impressive cycle life of 15 000 cycles as a rocking‐chair Zn‐ion battery anode.

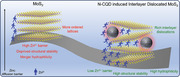

## 1 Introduction

The thrive to move beyond conventional lithium‐ion batteries (LIBs) in the energy storage sector has been gaining momentum recently, catalyzed by the serious safety concerns with organic electrolytes and lithium species, inferior resource availability, high cost of constituents (Li and Co‐containing cathodes), and environmental concerns from thermal runaway.^[^ ^1^ ^]^ Consequently, aqueous rechargeable batteries using water‐based electrolytes are gaining more importance due to their high safety, low manufacturing cost, and high ionic conductivity.^[^ ^2^ ^]^ Among multitudinous aqueous batteries, aqueous zinc‐ion batteries (AZIBs) show promising features, including high theoretical capacity (5855 mAh cm^−3^ and 820 mAh g^−1^), low‐cost of Zn‐metal (≈2 USD kg^−1^), high ionic conductivity (0.1 to 6 S cm^−1^), resource abundancy (79 ppm), two‐electron redox capability, facile fabrication, and high safety.^[^ ^3^ ^]^ More importantly, their low standard redox potential of −0.762 V (vs standard hydrogen electrode) allows their usage in aqueous electrolytes with mild acidic/basic mediums. Moreover, Zn metal is the only one among other alternatives (such as Li, Na, K, Mg, and Ca) that can be used directly in the aqueous electrolyte owing to its relatively high overpotential toward H_2_ evolution.^[^ ^4^ ^]^ Therefore, most of the ongoing research focuses on enhancing the capacity and durability of cathode materials while retaining Zn metal as the anode.^[^ ^5^ ^]^

However, there are several issues in using Zn metal directly as the anode, such as 1) dendrite formation, 2) H_2_ evolution, and 3) corrosion.^[^ ^6^ ^]^ Dendrite formation due to uneven stripping/platting of Zn ions may not only lower the Coulombic efficiency of the battery but also damage separators, eventually leading to short‐circuit. H_2_ evolution during charging can increase the pressure inside the cell, which can cause electrolyte leakage. Further, the insoluble by‐products (such as ZnO and Zn(OH)_2_) formed due to electrochemical corrosion can cover the active nucleation sites of the Zn anode surface and block the migration of electrons/ions in the interphase. Since all the above‐discussed drawbacks are interlinked to each other, they would affect and elevate each other.^[^ ^6c^ ^]^ Therefore, serious efforts are required to surmount these issues to meet the large‐scale application goal of AZIBs. Recent developments in the anode research follow two pathways: i) modifying Zn metal by surface/structural modification and alloying, and ii) replacing Zn metal with intercalation‐type materials.^[^ ^7^ ^]^ Although Zn metal modification can mitigate its inherent issues to some extent, the complete removal of existing bottlenecks with Zn anode remains questionable. In addition, the low gravimetric/volumetric capacity of AZIBs, caused by the low utilization depth of Zn‐based anodes, remains unresolved for practical scenarios.^[^ ^8^ ^]^ Therefore, the use of a “Zn‐metal free” or “rocking‐chair” configuration is considered as a promising solution. Moreover, the success history of LIBs also suggests that the “rocking‐chair” configuration can offer high performance and better safety.

The concept of “rocking‐chair” ZIBs was first introduced by Cheng et al.^[^ ^9^ ^]^ in 2016 by using Chevrel phase Mo_6_S_8_ as the anode, which delivered a maximum specific capacity of 55 mAh g^−1^ at 0.5 C and a good cycle life of ≈90% retention after 350 cycles. Thereafter, various anode materials such as transition metal oxides (MoO_3_, MoO_2_/carbon, H_2_Ti_3_O_7_, VO_2_, WO_3_/WC),^[^ ^10^ ^]^ transition metal chalcogenides (Na_0.14_TiS_2_, MoS_2_, CuS, Cu_2‐x_Se, Cu_7_Te_4_),^[^ ^11^ ^]^ metal bromides (Co‐BiOBr)^[^ ^12^ ^]^ and organic compounds (9, 10‐anthraquinone, perylene‐3, 4, 9, 10‐tetrecarboxylic diimide)^[^ ^13^ ^]^ were explored as the anode materials for AZIBs. However, the unsatisfactory energy density and limited choice of intercalation‐type materials need to be addressed further. Molybdenum disulfide (MoS_2_), a 2D layered transition metal chalcogenide with Mo sandwiched S atomic layers, is a highly promising intercalation‐type material for various energy storage systems, including AZIBs.^[^ ^14^ ^]^ Although MoS_2_ is widely explored as a cathode in AZIBs, its low discharge potential of 0.6 V (vs Zn/Zn^2+^) suggests that it might be more suitable as an anode when paired with a high‐potential cathode for “rocking‐chair” AZIBs. However, to date, very few studies have investigated its performance as an anode, which warrants further testing.

MoS_2_, as an intercalation‐type material for AZIBs, has offered limited specific capacity, lower rate performance, and limited cycle life because of its inferior electronic conductivity, large volume expansion, and hydrophobicity. Most importantly, insufficient interlayer spacing (6.15 Å between Mo‐Mo layers and 3.1 Å between S‐S layers) in comparison to the size of the hydrated Zn^2+^ ions (5.5 Å) results in a high Zn‐ion diffusion barrier, which seriously hinders their electrochemical performance.^[^ ^15^ ^]^ To mitigate these issues, researchers have implemented various strategies, which include i) structural engineering by synthesizing MoS_2_ with different structures, ii) interlayer engineering by expanding interlayer spacing, iii) defect engineering by creating vacancies, iv) phase engineering by introducing metallic 1T phase, and v) hybridization by introducing highly conductive components.^[^ ^16^ ^]^ Among these, expanding interlayer spacing is found to be more effective in various aspects as it can facilitate ion intercalation/deintercalation by effectively reducing the diffusion resistance^[^ ^14^, ^15^, ^16^ ^]^ and also the conversion of the semiconductive 2H phase into metallic 1T phase.^[^ ^14a^ ^]^ Apart from that, the introduction of foreign molecules during the synthesis of MoS_2_ can introduce various interlayer dislocations.^[^ ^17^ ^]^ Interlayer dislocations not only provide additional active sites for Zn‐ion storage beside lattice defects but also ensure out‐of‐plane charge transportation by connecting layers in the vertically stacked MoS_2_ lattice.^[^ ^18^ ^]^

In this foreground, the present study deals with a simple strategy for achieving increased interlayer spacing and rich lattice dislocations in MoS_2_ by the incorporation of Nitrogen‐doped carbon quantum dots (N‐CQDs). N‐CQDs have been chosen as the intercalating agent because of their rich surface functional groups and smaller particle sizes.^[^ ^19^ ^]^ Initially, N‐CQDs were prepared by a one‐step hydrothermal process, which was then added to the reaction mixture of MoS_2_ to obtain MoS_2_@N‐CQDs. The presence of N‐CQDs in the reaction mixture of MoS_2_ affects their nucleation and restacking process and results in rich defects (due to interlayer dislocations) and increased interlayer spacing of 0.8 nm. Benefiting from both, MoS_2_@N‐CQDs showed an excellent specific capacity of 258 mAh g^−1^ at 0.1 A g^−1^ and impressive cycle stability over 2000 cycles. Mechanistic insights into the Zn‐ion storage kinetics of interlayer dislocated MoS_2_ were unraveled by molecular dynamic (MD) simulations. Further, the rocking‐chair ZIBs with MoS_2_@N‐CQDs as anode and Zn_x_MnO_2_ as cathode could deliver a promising energy density of 120.3 Wh kg^−1^ and an excellent cycle life of 97% after 15 000 cycles. To the best of our knowledge, there are no other reports available on the N‐CQDs‐assisted interlayer expansion/dislocation of MoS_2_ for Zn‐ion battery applications.

## 2 Results and Discussion

### 2.1 Formation of MoS_2_@N‐CQDs

**Figure 1:**
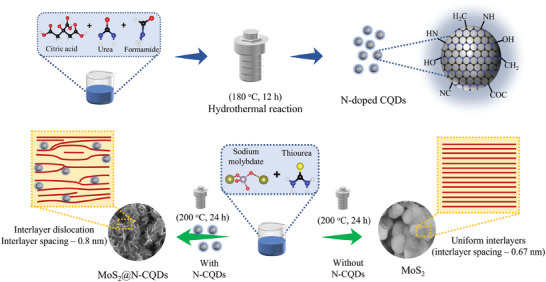
Schematic illustration of the synthesis of N‐CQDs and MoS_2_@N‐CQDs composite.

A two‐step solvothermal approach was used for the synthesis of MoS_2_@N‐CQDs, as schematically illustrated in **Figure** **1**. Initially, the chemical reaction between citric acid and urea in formamide solvent forms a eutectic system, which starts decomposing at 180 °C. This follows the formation of a graphitic carbon core by means of dehydration, polymerization, and carbonization.^[^ ^20^ ^]^ Meanwhile, the presence of urea facilitates N incorporation, forming N‐CQDs. In the second step, the H_2_S molecule from the hydrolyzed thiourea reduced the Mo^6+^ complex from Na_2_MoO_4_ to form Mo^4+^ ions. Its further reaction with S^2−^ ions under hydrothermal conditions produces MoS_2_. The typical synthesis mechanism of MoS_2_ is as follows.

(1) $$\begin{array}{ccc} & & 4Na_{2}\mathrm{Mo}O_{4}+15\mathrm{CS}{\left(NH_{2}\right)}_{2}+6H_{2}O\\ & & \;\to 4\mathrm{Mo}S_{2}+Na_{2}SO_{4}+6\mathrm{NaSCN}+24NH_{3}+9CO_{2} \end{array}$$

During hydrothermal treatment, the presence of N‐CQDs with rich surface functional groups alters the stacking of MoS_2_ layers by making electrostatic interaction with MoO_4_^2−^ ions during nucleation. This leads to the stabilization of the octahedral Mo center, which results in the formation of interlayer expanded MoS_2_ with a high 1T phase content. Moreover, since the N‐CQDs hinder the nucleation process, MoS_2_@N‐CQDs with rich layer dislocations are obtained.

### 2.2 Physicochemical Characterization of MoS_2_@N‐CQDs

The UV–visible spectrum of the N‐doped CQDs is shown in Figure S1a (Supporting Information). The absorption peak at 340 nm is attributed to the n → π^*^ electron transition of the C═O and C═N bonds of the N‐CQDs.^[^ ^21^ ^]^ The broad shoulder may arise from the n → σ^*^ electronic transitions of C─N and C─O bonds. The inset of Figure S1a (Supporting Information) shows the blue emission of N‐doped CQDs under a UV lamp having a wavelength of 253 nm. In Figure S1b (Supporting Information), the fluorescence spectrum of N‐CQDs under the excitation wavelength of 340 nm shows a strong emission peak centered at 480 nm. High‐intensity emission at higher wavelengths can be due to the presence of N‐containing moieties.^[^ ^22^ ^]^ Further, to identify the surface functionalities of N‐CQDs, Fourier transform infrared spectroscopy (FTIR) was performed and depicted in Figure S1c (Supporting Information). The broad absorption between 400 and 850 cm^−1^ corresponds to the aromatic C‐H bending vibrations and the peak at 1049 cm^−1^ corresponds to C─O─C stretching vibrations.^[^ ^23^ ^]^ The C─N stretching vibrations contribute to the absorption peaks at 1300 and 1384 cm^−1^,^[^ ^23a^ ^]^ while the strong peak centered at 1661 cm^−1^ is due to multiple absorptions from aromatic C═C bonds and carboxylic C═O bonds.^[^ ^24^ ^]^ Moreover, the minor absorption at 2880 cm^−1^ corresponds to ─CH_2_ vibrations,^[^ ^25^ ^]^ and the broad absorption between 2930 and 3540 cm^−1^ can be ascribed to the O─H, N─H, and C─H bond vibrations.^[^ ^23b^ ^]^ Therefore, FTIR analysis confirms that the N‐CQDs contain rich N and O‐containing functional groups, which are responsible for bonding with the Mo lattice.

**Figure 2:**
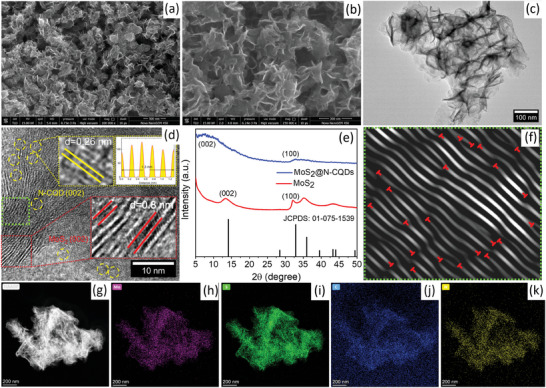
a,b) SEM images, c) TEM image, d) HRTEM image, and e) XRD pattern of the MoS_2_@N‐CQDs. f) iFFT of the green‐colored square region of (d), g) HAADF‐STEM image with the corresponding EELS mapping images for h) Mo, i) S, j) C, and k) N elements of MoS_2_@N‐CQDs.

Scanning electron microscope (SEM) images of MoS_2_@N‐CQDs (**Figure** **2**a,b) confirm the typical 2D sheet‐like morphology of the MoS_2_ nanostructures, which is homogeneous in almost the entire region without any agglomerations. In Figure S2 (Supporting Information), the thickness of the stacked layers of MoS_2_@N‐CQDs, as measured from various parts of the SEM images, lies in the range of 6–13 nm with an average thickness of 9.5 nm. SEM images of pristine MoS_2_ (Figure S3a–c, Supporting Information) also show sheet‐like morphology but with increased agglomerations and an average thickness (15.2 nm). Therefore, it can be inferred that the incorporation of N‐CQDs not only inhibits agglomeration but also reduces the number of stacking layers and hence the reduction of thickness of MoS_2_ nanosheets in the composite structure. In Figure 2c, transmission electron microscope (TEM) image again confirms the 2D ultra‐thin sheet‐like morphology of MoS_2_@N‐CQDs. To study the crystalline properties of the MoS_2_@N‐CQDs and confirm the presence of N‐CQDs, HRTEM analysis was performed. Figure S3d,e (Supporting Information) shows the TEM and HRTEM images of pristine MoS_2_ confirming their flake‐like morphology and an interlayer spacing of 0.67 nm, which corresponds to the (002) planes of 2H‐MoS_2_ (JCPDS: 01‐075‐1539). However, as shown in Figure 2d, the HRTEM image of MoS_2_@N‐CQDs demonstrates lattice planes with two different interlayer spacings, one of ≈0.8 nm, which can be assigned to interlayer expanded MoS_2_, and the other of ≈0.26 nm (highlighted with yellow circles in Figure 2d) representing the (100) planes of N‐CQDs.^[^ ^21^, ^26^ ^]^ To further confirm the increased interlayer spacing of MoS_2_@N‐CQDs, the X‐ray diffraction (XRD) pattern was analyzed. As in Figure 2e, the sharp diffraction peak at 13.28° corresponding to the (002) planes of the 2H‐MoS_2_ (JCPDS: 01‐075‐1539) is found to be downshifted in the case of MoS_2_@N‐CQDs and a broad diffraction hump between 5° to 15° could be observed. Such downshifting signifies an expansion in the interlayer spacings of MoS_2_ in the hybrid structure. Broad diffraction hump further suggests poor crystallinity of MoS_2_@N‐CQDs, which can be attributed to the interlayer dislocations and defects caused by the incorporation of N‐CQDs as well as a few‐layered structure of MoS_2_.^[^ ^27^ ^]^

Now, from the HRTEM images of MoS_2_@N‐CQDs, the extent of interlayer dislocations in MoS_2_ upon N‐CQDs incorporation was studied using inverse fast‐Fourier transform analysis (iFFT). Generally, dislocations are the kind of line defect that seems as a boundary between slid and non‐slip portions of the crystal.^[^ ^17^ ^]^ As it could connect the different planes of MoS_2_ and significantly alter their stress fields, dislocation plays a critical role in determining the physicochemical properties of the MoS_2_. Figure 2f shows the iFFT of the particular region from the HRTEM image of MoS_2_@N‐CQDs (dotted green box region in Figure 2d). It could be observed that numerous lattice dislocations were observed in MoS_2_@N‐CQDs (red marks in Figure 2d), while the uniformed lattice planes were observed in the case of pristine MoS_2_ (Figure S3f, Supporting Information). To get detailed insights, the areas corresponding to lattice dislocation were enlarged and classified in Figure S4 (Supporting Information). As shown in Figure S4 (Supporting Information), three major classes of interlayer dislocations can be observed depending on their distributions. Dislocation I is of the sandwich‐type caused due to the half layer slipping of the lattice plane and results in the reduction in the number of layers (three to two in defect “a” and four to three in defect “b”). The interaction of two close dislocation I leads to attraction, displacement, and dislocation dipole, resulting in dislocation type II. Here, free‐radical S atoms from the semi‐atomic lattice edge of the dislocation I attract with the disordered parallel lattice to reduce strain from dislocation II. Dislocation III is a kind of spiral dislocation with a relatively homogeneous strain that extends to a large area with less than half‐layer displacement. Figure S4 (Supporting Information) shows the co‐existence of all three kinds of dislocations in MoS_2_@N‐CQD. As discussed earlier, N‐CQDs have various surface functional groups, including N‐moieties, which have high affinity with the MoO_4_^2−^ ions by electrostatic interactions. This interaction will hinder the nucleation of MoS_2_ layers, leading to the formation of lattice‐dislocated MoS_2_ with increased interlayer spacing (Figure 2e). The rich lattice dislocations and amorphous nature of the MoS_2_@N‐CQDs upon N‐CQDs incorporation could also be confirmed by the selected area electron diffraction (SAED) analysis (Figure S5, Supporting Information), where MoS_2_@N‐CQDs showed broad circular bands while MoS_2_ has distinct lattice fringes corresponding to different lattice planes. Figure 2g–k depicts the high‐angle annular dark field scanning transmission electron microscopy (HAADF‐STEM) image of MoS_2_@N‐CQDs along with electron energy loss spectroscopy (EELS) mapping of Mo, S, C, and N elements, which confirms their homogeneous distribution throughout the composite structure. Meanwhile, the presence of only Mo and S elements in pristine MoS_2_ was also confirmed, as shown in Figure S6 (Supporting Information).

**Figure 3:**
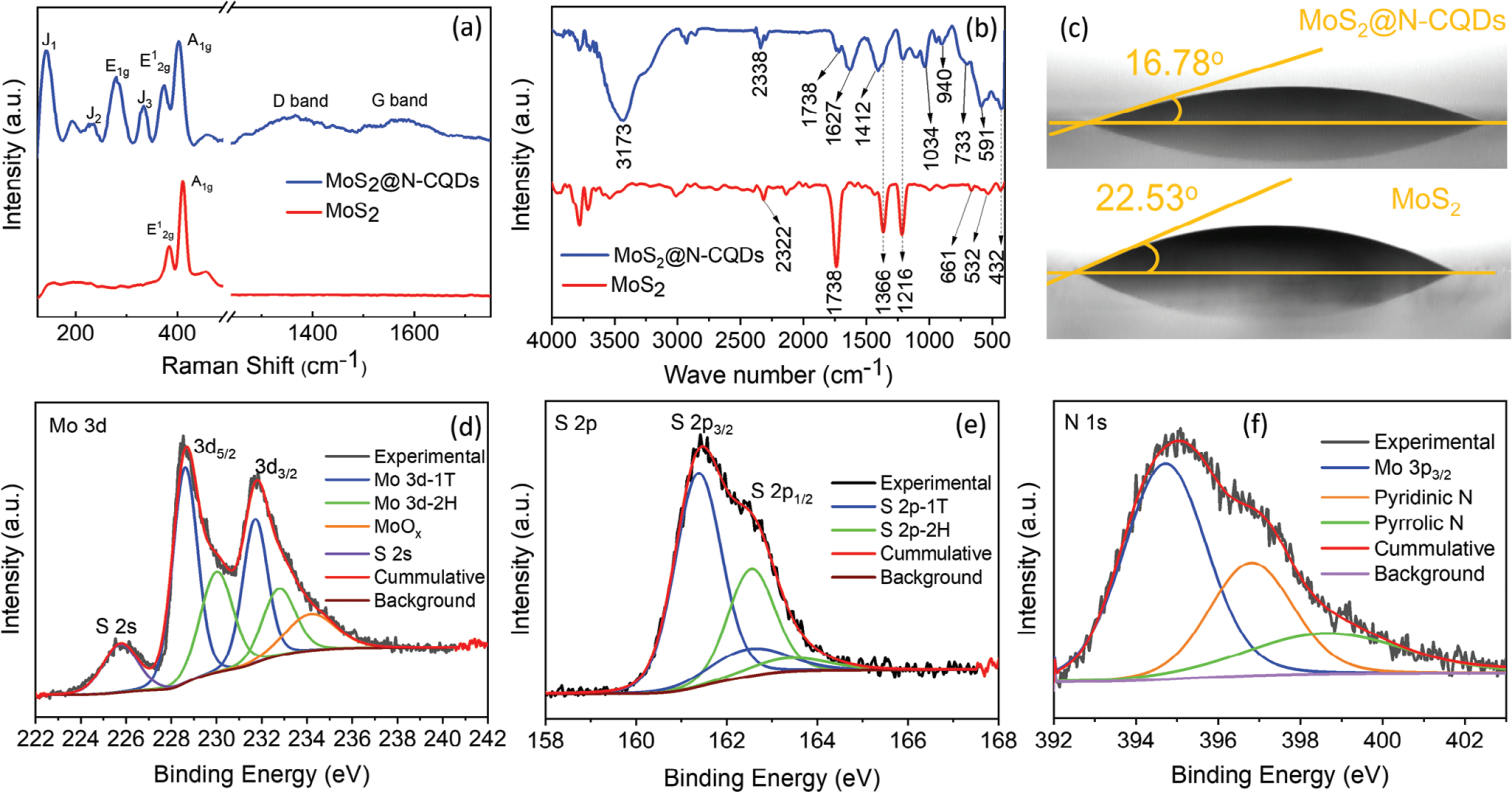
a) Raman, b) FTIR, and c) contact angle analysis results of MoS_2_ and MoS_2_@N‐CQDs. High‐resolution XPS spectra of MoS_2_@N‐CQDs with deconvoluted d) Mo 3d and e) S 2p and f) N 1s peaks.

To study the vibrational modes of the MoS_2_@N‐CQDs, the Raman spectroscopy technique was used (**Figure** **3**a). Pristine MoS_2_ showed two prominent peaks at 384 and 412 cm^−1^_,_ which are respectively attributed to the in‐plane $E_{2g}^{1}$ vibrations of S─Mo─S atoms and out‐of‐plane *A*_1*g*_ vibrations of the S atoms of the trigonal 2H phase of MoS_2_.^[^ ^28^ ^]^ For MoS_2_@N‐CQDs, four additional peaks at 140 (J_1_), 232 (J_2_), 280 (E_1_ _g_), and 334 (J_3_) cm^−1^ were observed, which are characteristic of the longitudinal acoustic phonon modes of metallic 1T MoS_2_.^[^ ^29^ ^]^ Therefore, the incorporation of N‐CQDs induces the formation of the octahedral 1T phase of MoS_2_. Moreover, the relative intensity ratio of $E_{2g}^{1}$ and *A*_1*g*_ was found to decrease from 2.76 in MoS_2_ to 1.26 in MoS_2_@N‐CQDs, suggesting an expansion in interlayer spacing.^[^ ^29^ ^]^ It is noteworthy that both $E_{2g}^{1}$ and *A*_1*g*_ bands of MoS_2_@N‐CQDs were red‐shifted by 9.8 and 8.1 cm^−1^, respectively; the red‐shifting of *A*_1*g*_ band was due to the decrease in interlayer interactions while the $E_{2g}^{1}$ band red‐shift represents an increase in electron concentration by N‐CQDs.^[^ ^30^ ^]^ More importantly, a larger red‐shift of $E_{2g}^{1}$ peak in comparison to *A*_1*g*_ peak implies the uniaxial strain in the MoS_2_ lattice,^[^ ^31^ ^]^ which can be the consequence of interlayer dislocations caused by N‐CQDs, as seen from the HRTEM images. The additional peak at 194 cm^−1^ in MoS_2_@N‐CQDs was due to the existence of different layers in MoS_2_.^[^ ^32^ ^]^ Moreover, the typical D (defective) and G (graphitic) bands of carbon observed at 1366 and 1581 cm^−1^, respectively, further confirm the existence of N‐CQDs in the MoS_2_ lattice.^[^ ^21b^ ^]^ Higher intensity of D band w.r.t G band (I_D_/I_G_ = 1.0251) signifies dominance of defective carbon resulting from the structurally embedded N‐atoms in the sp^2^ scaffold. Figure 3b compares the FTIR spectrum of MoS_2_ and MoS_2_@N‐CQDs. The absorption peaks at 432, 532. 661, 1216 and 1366 cm^−1^ of pristine MoS_2_ originated from the stretching vibrations of Mo─S bonds.^[^ ^33^ ^]^ For MoS_2_@N‐CQDs, the broad absorption between 400 and 820 cm^−1^ reveals the bending vibrations of the aromatic C─H bond of N‐CQDs (as also seen for N‐CQDs in Figure S1c, Supporting Information) along with Mo─S vibrations.^[^ ^23b^ ^]^ The new peaks of MoS_2_@N‐CQDs at 591, 733, 940, 1034, 1412, and 1627 cm^−1^ can be ascribed to C─S, C═S, C─O─S, C─C, O─H, and C═C vibrations, respectively,^[^ ^34^ ^]^ where the presence of C─S and C═S reveals strong interaction between MoS_2_ and N‐CQDs.^[^ ^34^ ^]^ Besides, the broad absorption centered at 3173 cm^−1^ is attributed to O─H, N─H, and C─H bonds from N‐CQDs.^[^ ^23b^ ^]^ Further, as better hydrophilicity of the electrode material plays a critical role in the electrochemical performance of AZIBs, contact angle analysis was performed using the sessile drop method (Figure 3c). The results signify that both MoS_2_ and MoS_2_@N‐CQDs are hydrophilic in nature with contact angles of 22.53° and 16.78°, respectively. Bettered hydrophilicity of MoS_2_@N‐CQDs can be due to the presence of different nitrogen and oxygen‐containing functional groups in N‐CQDs.^[^ ^35^ ^]^

The chemical states of different constituent elements of MoS_2_@N‐CQDs were investigated using X‐ray photoelectron spectroscopy (XPS) which confirms the presence of Mo, S, N, and C elements (Figure S7, Supporting Information). In Figure 3d, deconvolution of Mo 3d spectrum suggests the presence of Mo^4+^ 3d states (Mo 3d_5/2_ at 228.68 and Mo 3d_3/2_ at 231.74 eV) and Mo^6+^ 3d state (at 234.23 eV).^[^ ^36^ ^]^ Deconvolution also reveals the presence of 1T and 2H phases of MoS_2_, and the peaks for the dominant 1T phase can be seen downshifted by ≈1 eV as compared to their 2H counterparts, in agreement with other literature reports.^[^ ^14^, ^36^ ^]^ It is implied that the presence of N‐CQDs increased the electron density of Mo and S atoms, as also observed in Raman studies, which lowered their binding energy upon the increase in interlayer spacing.^[^ ^36^ ^]^ Amounts of 1T and 2H phases were calculated to be 56.4% and 37.6%, respectively, in the composite structure. However, the presence of Mo^6+^ with 5.9% concentration arose from the surface oxidation of MoS_2_ during the XPS study.^[^ ^37^ ^]^ In Figure 3e, the deconvolution of S 2p further confirms the polymorphic nature of MoS_2_ in the composite structure. The High‐resolution N 1s spectrum in Figure 3f shows two characteristic peaks of N at 396.8, and 398.5 eV, corresponding to the pyridinic N and pyrrolic N of the N‐CQDs,^[^ ^10b^ ^]^ while the strong peak at 394.7 eV corresponds to Mo 3p_3/2_.^[^ ^38^ ^]^ Deconvolution of Mo 3d and S 2p peaks for pristine MoS_2_ (Figure S8, Supporting Information) suggests the co‐existence of both 1T and 2H phases, however, with a relatively lower 1T phase concentration (49.7%) as compared to the same in MoS_2_@N‐CQDs hybrid, thus substantiating the positive traits of N‐CQDs in forming and stabilizing the 1T phase.

From the above morphological and structural characterizations of MoS_2_@N‐CQDs, it is evident that the incorporation of N‐CQDs momentously alters the physical (lattice defects, number of layers, crystal phase) and chemical (composition, functional groups, and wettability) properties of the MoS_2_.

Further, to calculate the N‐CQDs amount in MoS_2_@N‐CQDs, thermogravimetric analyses (TGA) were performed (Figure S9, Supporting Information). Initial weight loss of 8.44% up to 120 °C was caused by the evaporation of surface adsorbed water and volatile impurities in the sample, while the second weight loss of 18.66 wt. % between 120 and 385 °C manifests the removal of structural water between the lattice planes of MoS_2_.^[^ ^39^ ^]^ This corresponds to the 1.5 H_2_O molecules per MoS_2_ unit (i.e., MoS_2_.1.5H_2_O). The third mass loss above 385 °C could be ascribed to the oxidation of MoS_2_ as per Equation (2) and the conversion of carbon into CO_2_.^[^ ^26^ ^]^

(2) $$2\mathrm{Mo}S_{2}+7O_{2}\to 2\mathrm{Mo}O_{3}+4SO_{2}$$

From the TGA analyses and using Equation S1 (Supporting Information), the amount of MoS_2_ in MoS_2_@N‐CQDs was found to be 75.73%, which gives the N‐CQDs an amount to be 24.27% in the composite.

### 2.3 Electrochemical Characterization of MoS_2_@N‐CQDs in Zn‐MoS_2_ Batteries

**Figure 4:**
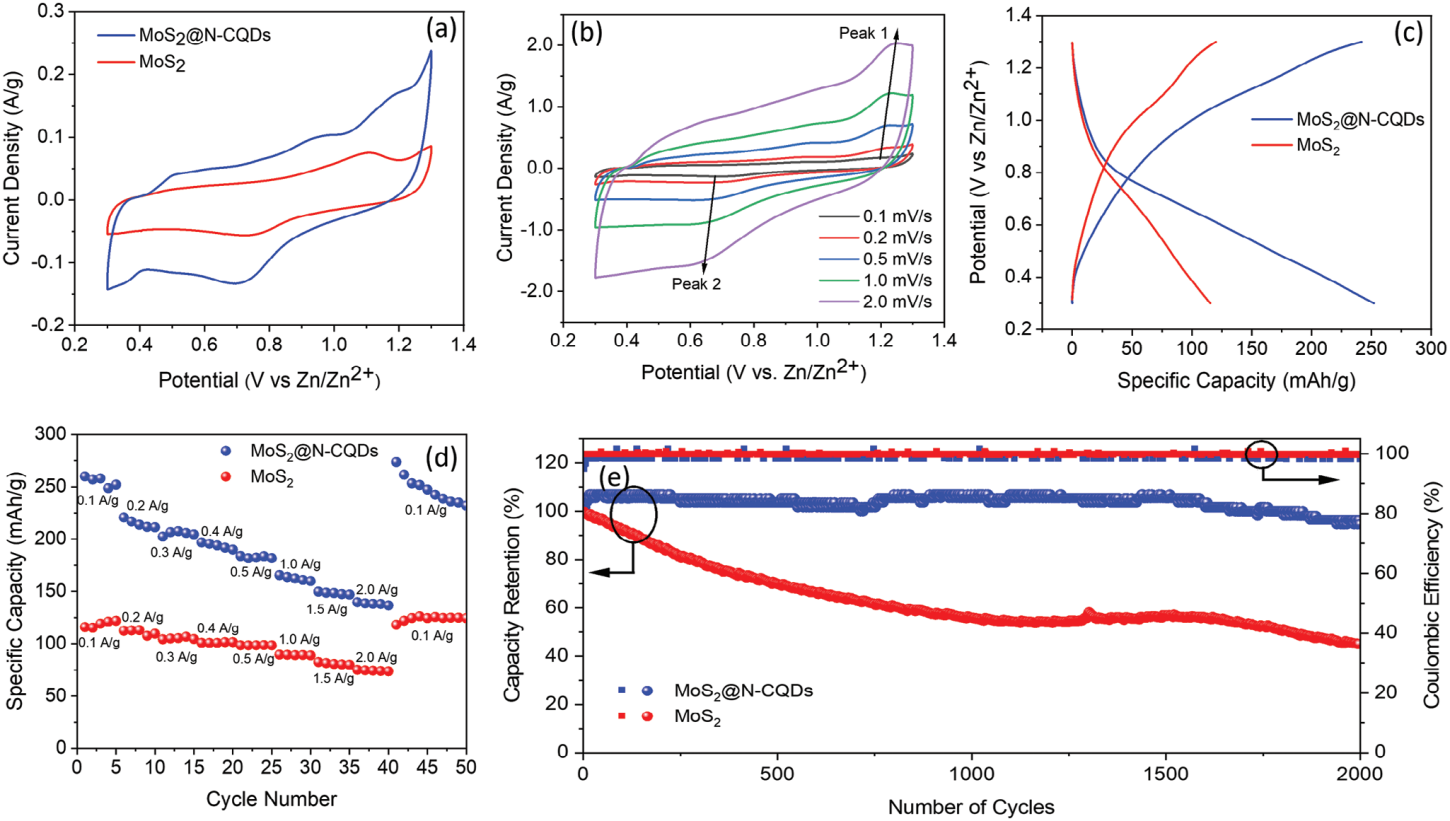
a) Comparison of CV curves of MoS_2_ and MoS_2_@N‐CQDs at 0.1 mV s^−1^, b) CV curves of MoS_2_@N‐CQDs at various scan rates, c) GCD curves of both the electrode materials at 0.1 A g^−1^, comparison of MoS_2_ and MoS_2_@N‐CQDs in terms of d) rate capability (for 50 cycles) and e) cyclic performance (for 2000 cycles).

The Zn‐ion storage performance of MoS_2_ and MoS_2_@N‐CQDs was studied in half‐cell configuration using Zn foil as counter as well as reference electrode and 3 m Zn(OTf)_2_ as electrolyte. **Figure** **4**a compares the cyclic voltammetry (CV) curves of MoS_2_ and MoS_2_@N‐CQDs within a potential window of 0.3−1.3 V at 0.1 mV s^−1^. The CV curve of MoS_2_ showed two main redox peaks around 1.1 V (oxidation Mo^4+^ to Mo^6+^) and 0.75 V (reduction of Mo^6+^ to Mo^4+^), corresponding to the deintercalation and intercalation of Zn‐ions, respectively.^[^ ^40^ ^]^ For MoS_2_@N‐CQDs, the oxidation and reduction peaks were found to be almost at similar voltages to that of pristine MoS_2_. However, the increased integral area of the CV curve for MoS_2_@N‐CQDs suggests its improved Zn‐ion storage capacity. Notably, in the CV of MoS_2_@N‐CQDs, an additional hump could be observed at the lower potential (0.3 V), which can be due to the additional intercalation of Zn‐ions induced by the alteration in the order/disorder structure of the Zn^2+^/H_2_O superlattice in MoS_2_@CQDs.^[^ ^39^, ^41^ ^]^ This further revealed that the interlayer dislocation caused by the introduction of N‐CQDs significantly affects the electrochemical performance of MoS_2_. Figure 4b shows the CV curves of MoS_2_@N‐CQDs at different scan rates. The shape of the CV curves was almost similar, even at higher sweep rates, which signifies the small polarization voltage of the material. However, as the scan rates increased from 0.1 to 2 mV s^−1^, there was a slight shift in the anodic and cathodic peaks toward higher and lower potentials, respectively, indicating a slight increase in the diffusion resistance of MoS_2_@N‐CQDs.^[^ ^42^ ^]^ The corresponding specific capacities of MoS_2_@N‐CQDs were calculated to be 239, 216, 188, 168, and 147 mAh g^−1^ at 0.1, 0.2, 0.5, 1, and 2 mV s^−1^, respectively, which were significantly higher as compared to the pristine MoS_2_.

For further understanding of the charge storage capacity, galvanostatic charge‐discharge (GCD) profiles of the synthesized materials were measured and compared at 0.1 A g^−1^, as shown in Figure 4c. It is noteworthy that the absence of distinct plateau regions in the charge/discharge profiles for both materials implies the single‐phase continuous (de)intercalation of Zn^2+^ ions between the lattice planes of MoS_2_ as well as its pseudocapacitive charge storage process in line with the CV analyses.^[^ ^39b^ ^]^ It can be further observed that at 0.1 A g^−1^ the specific capacity increased from 120 to 258 mAh g^−1^ upon incorporating N‐CQDs into the MoS_2_ matrix supplementing improved charge storage performance of MoS_2_@N‐CQDs. The rate performance of the materials was also analyzed by step‐wise ramping the current densities from 0.1 to 2 A g^−1^ (Figure 4d). The specific capacities for the initial cycles of MoS_2_@N‐CQDs at 0.1, 0.2, 0.3, 0.4, 0.5, 1, 1.5, and 2 A g^−1^ were calculated to be 258, 220, 202, 197, 183, 165, 150 and 134 mAh g^−1^, respectively. After 40 cycles, the MoS_2_@N‐CQDs have almost recovered their initial specific capacity at 0.1 A g^−1^. However, capacity decreases afterward and settles during the following cycles. In contrast, much lower specific capacity values of 116, 112, 104, 101, 98, 89, 82, and 75 at respective 0.1, 0.2, 0.3, 0.4, 0.5, 1, 1.5, and 2 A g^−1^ were observed for pristine MoS_2_, further suggesting efficacy of N‐CQDs in Zn‐ion storage. The possible charge storage reaction between MoS_2_@N‐CQDs and Zn‐metal anode is depicted as follows;

(3) $$\mathrm{Cathode}:\mathrm{xZ}n^{2+}+2xe^{-}+\mathrm{Mo}S_{2}↔Zn_{x}\mathrm{Mo}S_{2}$$

(4) $$\mathrm{Anode}:\mathrm{xZ}n^{2+}+2xe^{-}↔\mathrm{xZn}$$

As per Faraday's equation (Equation S2, Supporting Information), the number of Zn^2+^ ions (*x*Zn) intercalated into the MoS_2_@N‐CQDs lattice was calculated to be 0.77 at 0.1 A g^−1^, which was actually >≈0.41 as calculated for pristine MoS_2_, thus substantiating the effect of N‐CQDs in facilitating Zn‐ion movement through interlayer expanded MoS_2_ and enhance storage capacity.

Figure 4e depicts the cycling performance of MoS_2_ and MoS_2_@N‐CQDs at 2 A g^−1^. It could be observed that MoS_2_ showed continuous degradation in the specific capacity, and after 2000 cycles, only 45% of the initial capacity was retained, which indicates structural instability of MoS_2_ upon continuous Zn‐ion intercalation/deintercalation processes and the possibility of Zn dendrite formation. On the other hand, MoS_2_@N‐CQDs showed excellent cycling stability with a capacity retention of 94.5% after 2000 cycles, which supports their durable structure aided by N‐CQDs addition. It can be quite understandable that the interlayer expansion of (≈8 Å) of MoS_2_ due to the presence of N‐CQDs is much higher than the size of the hydrated Zn^2+^ ions (5.5 Å), and hence, could facilitate the Zn‐ion intercalation/intercalation processes without causing much strain in the MoS_2_ lattice structure. Therefore, MoS_2_@N‐CQDs demonstrated much better electrochemical cycle stability as compared to pristine MoS_2_. The overall coulombic efficiencies for both materials were found to be almost stable and close to 100%. Such Zn‐ion storage performance of MoS_2_@N‐CQDs is found to be much better than many other similar literature works, as summarized in Table S1 (Supporting Information). Further, to understand the underlying reason behind the bettered performance of MoS_2_@N‐CQDs in comparison to MoS_2_, the electronic conduction behavior of the electrode materials was studied through electrochemical impedance spectroscopy (EIS) before and after cycling tests, and the results are depicted in Figure S10 (Supporting Information). The Nyquist plots were fitted with the equivalent circuit model and different resistance components obtained were summarized in Table S2 (Supporting Information). It is noteworthy that initially, the MoS_2_@N‐CQDs showed less equivalent series resistance (R_s_) and charge transfer resistance (R_ct_) of 2.77 ohm and 4.1 ohm, respectively, in comparison to pristine MoS_2_ having high R_s_ and R_ct_ values of 4.73 ohm and 6.24 ohm, respectively, which supports better charge storage of the composite electrode. After 2000 cycles, the resistance values of both materials increased, however, the change was less in the case of MoS_2_@N‐CQDs, which further signifies its better structural stability during cycling. Moreover, SEM‐EDS analysis was performed for the post‐cycled MoS_2_@N‐CQDs sample to analyze its morphological changes during cycling, as depicted in Figure S11a,b (Supporting Information). A sheet‐like morphology of the MoS_2_@N‐CQDs (marked by yellow colored arrows in Figure S11b (Supporting Information) suggests the good stability of the active material. However, there are some agglomerations observed in the structure, which can be due to the physical mixing of acetylene black and PVDF during the slurry casting process.^[^ ^43^ ^]^ EDS mapping in Figure S11c (Supporting Information) confirms the uniform distribution of Zn, along with Mo, S, C, and N in the MoS_2_@N‐CQDs even after 2000 charge/discharge cycles. Figure S12a,b (Supporting Information) shows the comparison of CV and GCD curves of all the synthesized samples in which MoS_2_@N‐CQDs with 6 mL of N‐CQDs showed enlarged CV area and higher specific capacity than others. Rate performance studies from Figure S12c (Supporting Information) reveal that both specific capacity and rate capability increase with the concentration of N‐CQDs and reach a maximum for a 6 mL N‐CQDs sample. The increase in concentration of N‐CQDs leads to the increase in lattice dislocations and interlayer spacing which results in the increased specific capacity for MoS_2_@N‐CQDs with 6 mL N‐CQDs. However, the further increase in the concentration (8 mL) resulted in a decline in both specific capacity and rate capability. This can be attributed to the high defectiveness of MoS_2_@N‐CQDs structure results from the high concentration of N‐CQDs, which may deploy the conductivity of the material. A similar trend was observed for the cycling studies (Figure S12d, Supporting Information) in which MoS_2_@N‐CQDs with 2, 4, 6, and 8 mL of N‐CQDs have shown 48.6%, 63%, 94.5%, and 87.2%, respectively

### 2.4 Electrochemical Kinetics Analysis of MoS_2_@N‐CQDs

**Figure 5:**
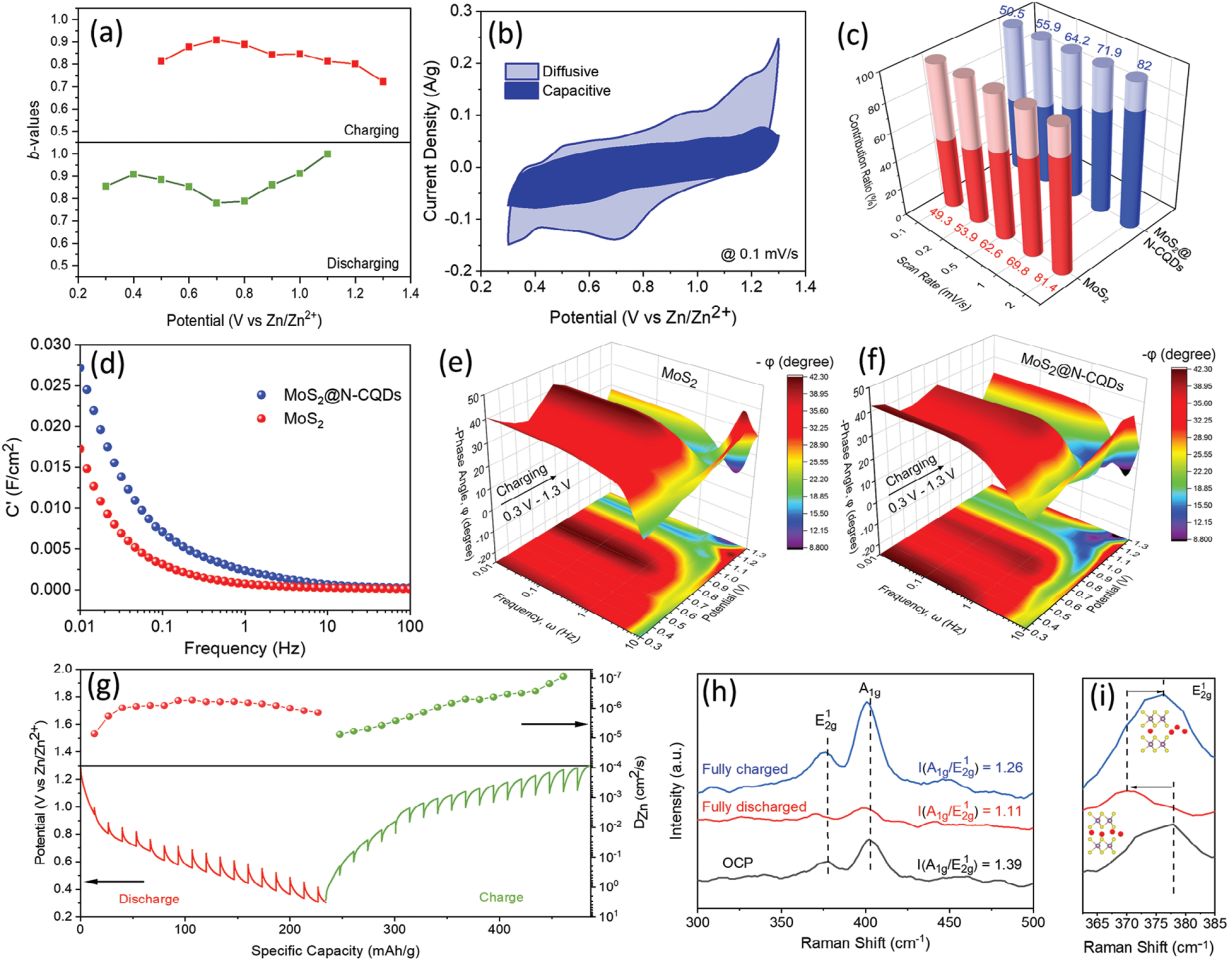
a) variation of *b*‐values at different charge/discharge potentials and b) segregation of capacitive and diffusive Zn^2+^ storage contributions at 0.1 mV s^−1^ of MoS_2_@N‐CQDs, c) comparison of capacitive and diffusive contributions of MoS_2_ and MoS_2_@N‐CQDs at different scan rates; d) 2D Bode plot and 3D Bode impedance plot of e) MoS_2_ and f) MoS_2_@N‐CQDs during charging. g) GITT profile of MoS_2_@N‐CQDs and its Zn^2+^ diffusion coefficient values calculated at 0.1 A g^−1^; h) comparison of normalized intensities of ex situ Raman spectra of MoS_2_@N‐CQDs at various charge/discharge states and i) its enlarged version of $E_{2g}^{1}$ peak.

To understand the Zn‐ion storage mechanism further, the contribution from the reaction‐controlled (diffusive) and reaction‐independent (capacitive) processes to the overall specific capacity of MoS_2_@N‐CQDs has been evaluated using Dunn's method as explained in detail in the supporting information. In theory, *b*‐value in the Formula S3 (Supporting Information) reflects the kinetics of the electrochemical mechanism with *b* = 0.5 and *b* = 1 referring to diffusion‐controlled and capacitive‐dominated processes, respectively.^[^ ^44^ ^]^ Herein, using the CVs in Figure 4b, the *b*‐values at each potential were calculated from the slope of the log (current) versus log (scan rate) graph (Figure S13, Supporting Information) and are plotted in **Figure** **5**a. The lowest *b* value of 0.72 at 1.3 V during the charging process corresponds to the oxidation of Mo^4+^, while during discharging, *b* = 0.78 at 0.7 V signifies the reduction process of Mo^6+^. Figure 5a further unveils that the *b*‐values lie between 0.7 and 1, thus implying the contributions of both diffusion‐controlled and capacitive‐controlled processes. This observation further stimulated us for a quantitative evaluation of their contributions to the total charge storage as per Equations S3 and S4 (Supporting Information). As shown in Figure 5b, even at a lower scan rate of 0.1 mV s^−1^, 50.5% of the total capacity of MoS_2_@N‐CQDs resulted from the adsorption/desorption‐based capacitive charge storage mechanism. This can be understood very easily in terms of the free flow of Zn^2+^ ions through the expanded spacings between 2D MoS_2_ layers owing to the presence of N‐CQDs followed by redox‐based and capacitive charge storage in MoS_2_, N moieties of N‐CQDs, and CQDs itself. As the scan rate was increased from 0.1 to 2 mV s^−1^ (Figure 4c), the capacitive contribution dominated over the diffusive contribution because of the inaccessible active sites in the bulk of MoS_2_@N‐CQDs to the faster‐moving Zn‐ions, forcing charge storage predominantly in the surface and near‐surface regions of the electrode material. A similar trend was observed in MoS_2_ also (as from Figure 5c; Figure S14, Supporting Information). However, their capacitive contribution is lesser (49.3% at 0.1 mV s^−1^) than that of MoS_2_@N‐CQDs. Therefore, it is evident that the incorporation of N‐CQDs significantly increased the capacitive contribution of MoS_2_. Further, in‐depth kinetics of both the materials were studied by 2D Bode and 3D Bode analysis. For that, EIS analysis was performed at different potentials (0.3 to 1.3 V) during the charging and discharging processes. Figure 5d shows the 2D bode plot at open circuit potential (OCP), which depicts the real capacitance (C’) (calculated from Equation S5, Supporting Information) as a function of frequency (ω). MoS_2_@N‐CQDs show a higher C’ value in comparison with MoS_2_, which signifies a greater number of Zn‐ion (de)intercalations and faster electrochemical kinetics.^[^ ^45^ ^]^ Figure 5e, f displays the 3D Bode impedance plot of MoS_2_ and MoS_2_@N‐CQDs, which mapped the change in phase angle (φ) with respect to different frequencies and potentials. Here φ = 90°, 45°, and 0° respectively, signifies the pure capacitive, diffusive, and pure resistive behaviors charge storage kinetics. Since Faraday impedance has the resistive characteristic during electrochemical reactions, φ tends to 0° at the potentials where redox reactions take place.^[^ ^45a^ ^]^ For pristine MoS_2_ (Figure 5e), during charging, φ values approaching 0° between 1.05 to 1.25 V in the medium and high‐frequency regions, which corresponds to its oxidation potential.^[^ ^40^ ^]^ A similar trend is observed for MoS_2_@N‐CQDs, as of Figure 5f; however, φ tending toward a lower angle is smaller in comparison with MoS_2_. This confirms the faster kinetic reaction in the MoS_2_@N‐CQDs by the increased capacitive ion reactive ion contribution.^[^ ^45a^ ^]^ Similarly, during discharging (Figure S15, Supporting Information), the small trough is observed in the range of 0.7 V, which corresponds to the reduction of Mo^6+^ to Mo^4+^. The faster reaction kinetics of MoS_2_@N‐CQDs could be noticed from the slight increase in φ. Therefore, Dunn's method, 2D Bode, and 3D Bode analyses clearly revealed that N‐CQDs elevated the electrochemical kinetics MoS_2_ by the increased capacitive contribution.

Further, a galvanostatic intermittent titration test (GITT) was performed to investigate the diffusion coefficient of the materials at different charge/discharge states (Figure 5g). As calculated using Equation S6 (Supporting Information), the GITT analyses of pristine MoS_2_ reveal a small diffusion coefficient (D_Zn_) of 10^−9^ to 10^−12^ cm^2^ s^−1^ in between 0.3 to 1.3 V, as shown in Figure S16 (Supporting Information). Notably, the D_Zn_ of MoS_2_@N‐CQDs was found to lie within the range of 10^−6^ to 10^−8^ cm^2^ s^−1^, which suggests a much faster diffusion kinetics facilitating Zn^2+^ ion movement even at slower scan rates and results in a significant proportion of capacitive charge in the total charge stored by the composite electrode. Further, the obtained values of MoS_2_@N‐CQDs were also found to be higher than the D_Zn_ values of other MoS_2_‐based pristine and composite electrode materials reported elsewhere (Table S1, Supporting Information).

To get deeper insights into the Zn‐ion storage mechanism of MoS_2_@N‐CQDs, ex situ Raman spectra were recorded at OCP, fully‐discharged state, and fully‐charged state and analyzed (Figure 5h). At OCP, typical Raman modes of MoS_2_, such as $E_{2g}^{1}$ and *A*_1*g*_ at 378 and 402 cm^−1^ from the in‐plane S─Mo─S vibrations and out‐of‐plane Mo─S vibration were observed, and their intensity ratio (I(*A*_1*g*_)/I($E_{2g}^{1}$)) was calculated to be 1.39. After the first discharging (at 0.3 V), a decrease in the intensity ratio to 1.11 was observed, which can be attributed to the increase in interlayer spacing during the intercalation of Zn^2+^ ions into MoS_2_. Further, at the fully charged state, the intensity ratio was again found to increase to 1.26, suggesting structural reversibility of MoS_2_ upon Zn^2+^ deintercalation. Interestingly, apart from the intensity variation, a clear peak shift was also evident upon discharging and charging (Figure 5i). The red‐shift shift of $E_{2g}^{1}$ and *A*_1*g*_ peaks, specifically with larger shifts of $E_{2g}^{1},$ again signifies the dominance of uniaxial strain due to Zn^2+^ intercalation.^[^ ^31^ ^]^ From these observations, it can be argued that, upon intercalation of Zn^2+^ ions, the existing dislocation‐induced lattice strain increased further, which provided additional specific capacity, as observed as an additional peak in the CV curve at 0.3 V (Figure 4a). At the fully charged state, the peaks were blue‐shifted again due to reduced strain effects upon the deintercalation of Zn^2+^ ions. Importantly, both the peak positions and relative peak intensities were not completely regained at the fully charged state, which infers that not all the Zn^2+^ ions were deintercalated from the MoS_2_@N‐CQDs planes. From the ex‐situ Raman results, it can be argued that although there is a massive change in the local structure of the MoS_2_ during the intercalation process, the changes were almost reversible, which is attributed to the larger interlayer spacing in the MoS_2_ lattice aided by the presence of N‐CQDs. This further helps in the improved cyclic stability of MoS_2_@N‐CQDs by overcoming the volume expansion‐related issues during cycling. Therefore, the ex‐situ Raman analyses of MoS_2_@N‐CQDs have substantiated the efficacy of N‐CQDs in holding the structural integrity of MoS_2_ during long‐term charge‐discharge steps.

Based on the charge storage kinetic studies and ex situ Raman analyses, Zn^2+^ storage mechanisms for MoS_2_ and MoS_2_@N‐CQDs can be explained with the help of the schematics in Figure S17 (Supporting Information). Better crystalline MoS_2_, having a larger number of ordered lattices but with a limited interlayer spacing of 0.67 nm, offers a kinetic barrier toward the movement of hydrated Zn^2+^ ions because of their large size of 0.55 nm. Therefore, lattices of MoS_2_ undergo large volume variations during intercalation (discharge) and deintercalation (charge) processes and result in an ever‐decaying storage capacity during the prolonged charge/discharge cycles. Moreover, less number of Zn^2+^ active sites and poor hydrophilicity of MoS_2_ cause limited capacity. On the other hand, MoS_2_@N‐CQDs are rich in interlayer dislocations and have increased interlayer spacing (0.8 nm) owing to the presence of N‐CQDs. Higher interlayer spacing creates much less hindrance for Zn^2+^ ion movement, while abundant interlayer dislocations offer more storage sites, resulting in higher capacities even at higher current densities. Also, lattice dislocations can effectively accommodate the volume variations during the intercalation/deintercalation processes and hence, a bettered cyclic stability of the material. Furthermore, the high hydrophilicity of the N‐containing functional groups in N‐CQDs improved the wettability of the material, which resulted in faster diffusion kinetics and hence, a large capacitive contribution toward the total storage capacity of MoS_2_@N‐CQDs.

### 2.5 Mechanistic Understanding of Zn‐Ion Storage Through MD Simulations

**Figure 6:**
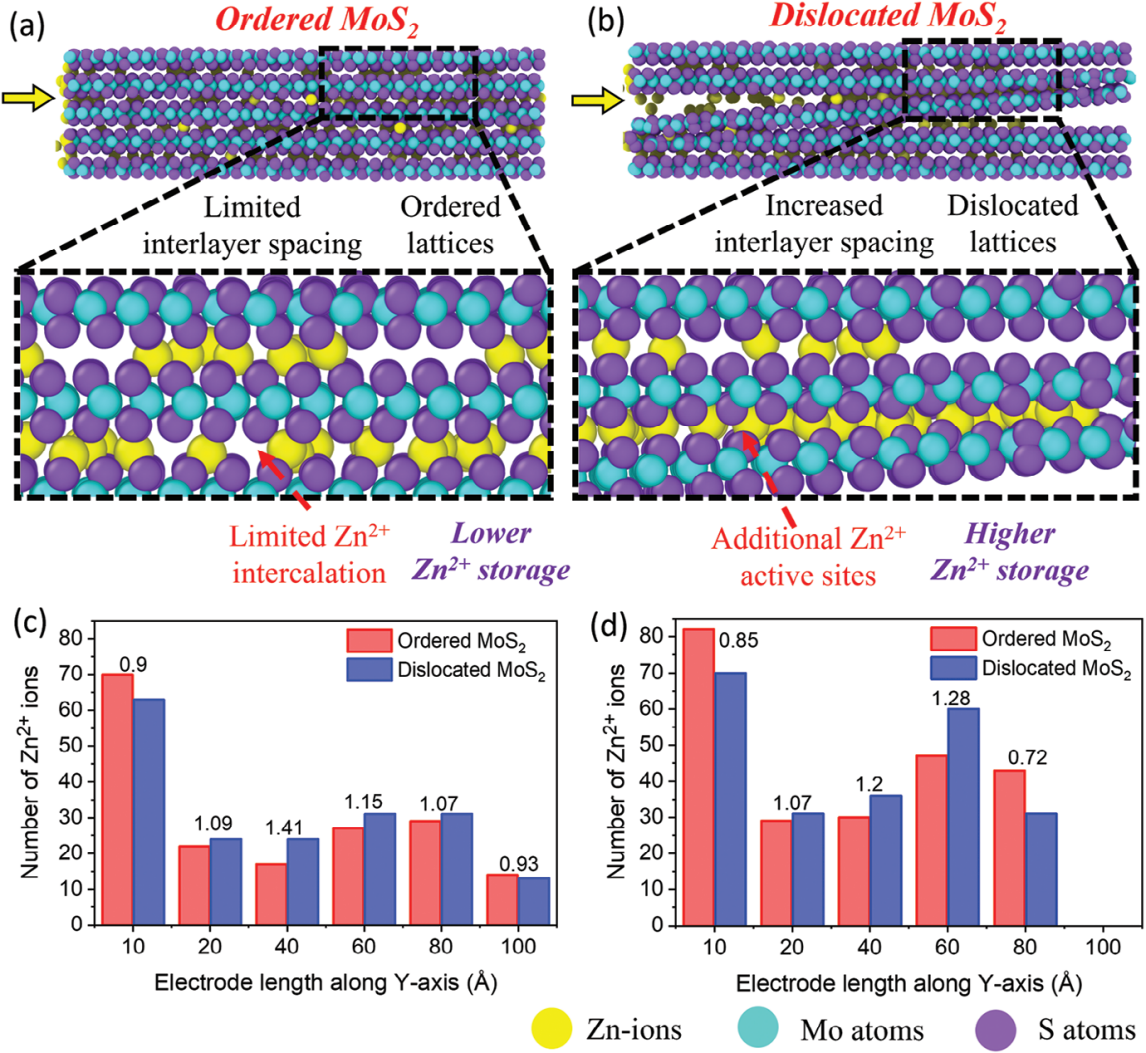
Snapshot of Zn‐ion storage inside a) ordered MoS_2_ and b) dislocated MoS_2_ electrodes at low current density. Quantitative MD simulation results for both the electrodes at c) low and d) high currents (values indicate the ratio of Zn ions in dislocated MoS_2_ to ordered MoS_2_).

To further unveil the role of interlayer dislocations in increasing the Zn‐ion storage performance on an atomic scale, MD simulations were performed. Here, four cases are considered where the first two dealt with Zn‐ion interaction (discharging phase of ZIB) with the ordered MoS_2_ electrode at lower and higher currents, and the rest dealt with dislocated MoS_2_ with lower and higher currents. Figure S18 (Supporting Information) depicts the MD simulation for the intercalation of Zn‐ions in ordered MoS_2_ and dislocated MoS_2_ electrodes at different time scales while applying low currents. At time step 1 (zero current state), few Zn‐ions are adsorbed on the surface of the MoS_2_ layers; as the current is applied, ions start intercalating into the interlayers of MoS_2_ in the subsequent time steps. It is visualized clearly that the Zn‐ions are moving in the adjacent layers of ordered MoS_2_ by one after another in a constrained way, because of their inferior interlayer spacing. However, the enlarged interlayer spacing at the dislocated site allows the intercalation of the stream of Zn‐ions in case of dislocated MoS_2_. This could contribute to the increased specific capacity of the MoS_2_@N‐CQDs. After complete intercalation at a lower current (**Figure** **6**a), it could be clearly visualized that ordered MoS_2_ has shown a lesser number of intercalated Zn‐ions in comparison with the dislocated MoS_2_. Moreover, it could also be seen from Figure 6b that there is a clustering of Zn‐ions in the dislocation sites, confirming that dislocations were acting as additional active sites for Zn‐ion storage. This can be quantitatively confirmed from Figure 6c, where it can be seen that the number of Zn‐ions on dislocated MoS_2_ at the electrode length of 20–80 Å (which corresponds to the dislocation sites) is higher than that of ordered MoS_2_. Therefore, it is evident that the introduction of lattice dislocations from N‐CQDs significantly elevates the Zn‐ion storage of MoS_2_ by the increased interlayer spacing and dislocation‐induced active storage sites. It is to be noted that the difference in the number of intercalated Zn‐ions in ordered and dislocated MoS_2_ is lesser in comparison with the difference in their specific capacity from the experimental results. This is because the sample size taken for MD simulations is much smaller (5 MoS_2_ layers with single dislocation) than the practical scenario. Figure S19 (Supporting Information) shows the snapshots of simulated ordered and dislocated MoS_2_ at a high current. As the charge values increase (increase in current density), the Zn‐ions get accelerated and lose the capacity to move uniformly and intercalate completely in the MoS_2_ lattice. This leads to the partial intercalation of Zn‐ions in MoS_2_, as seen in Figure S19a (Supporting Information). In the case of dislocated MoS_2_ (Figure S19b, Supporting Information), the clustering of Zn‐ions at the dislocation sites depicts the blockage of Zn‐ion transportation at the higher currents. From the quantitative simulation graph (Figure 6d), we can clearly see that the ratio of the number of Zn‐ions of dislocated MoS_2_ to ordered MoS_2_ (N_2_/N_1_) at 60 Å (corresponds to the neck of dislocation site) is higher at high current (N_2_/N_1_ = 1.28) than that of low current (N_2_/N_1_ = 1.15). This suggests the clustering of Zn‐ions at the dislocation sites. Further, while moving from 60 to 80 Å, the N_2_/N_1_ ratio decreased drastically from 1.07 to 0.72, revealing the blockage of Zn‐ion transportation at the dislocation sites. This could be correlated with the inferior rate capability of MoS_2_@N‐CQDs compared to MoS_2_. Further, such blockage of Zn‐ion movement at a high current could also be the reason for continuous decay in specific capacity at a low current (0.1 A g^−1^) during the rate reversibility test in Figure 4d. Due to the blockade created at higher currents at the dislocation sites, Zn ions may not fully access all the electrochemically active sites of MoS_2_@N‐CQDs when the current returns to lower values. This results in a gradual capacity decay during subsequent cycling. From the MD simulation results, we can conclude that i) the increased interlayer spacing in dislocated MoS_2_ allows more Zn‐ion transportation, and the dislocation sites store more Zn‐ions by acting as the active sites, ii) at high current, Zn‐ions could not able to intercalate completely in the MoS_2_ lattice results in decreasing capacity, iii) the dislocation sites in dislocated MoS_2_ are blocking the Zn‐ion transportation at high current, results in the decreased rate performance than ordered MoS_2_.

### 2.6 Full‐Cell Characterization of MoS_2_@N‐CQDs//Zn_x_MnO_2_

Inspired by the high Zn‐ion storage performance in half‐cell configuration, MoS_2_@N‐CQDs was further explored as an anode for Zn‐free rocking‐chair ZIBs with Zn_x_MnO_2_/CC as cathode, for the first time as of our knowledge. Zn_x_MnO_2_ was particularly chosen here because of its high plateau potential, inbuilt Zn in its structure, high specific capacity, and easy synthesis procedure.^[^ ^46^ ^]^ Here, Zn_x_MnO_2_ was synthesized using a previously reported hydrothermal method and characterized through XRD and SEM‐EDS techniques. Figure S20a (Supporting Information) depicts the XRD pattern where all the diffraction peaks are well indexed with the standard JCPDS (No. 44–0141) pattern of Zn_x_MnO_2_, confirming good crystallinity. However, the additional broad peak around 26° corresponds to the (002) plane of the carbon cloth (CC). As in Figure S20b,c (Supporting Information), the SEM characterizations show that Zn_x_MnO_2_ has a uniform rod‐like morphology with a diameter of ≈65 nm. It is also evidenced that Zn_x_MnO_2_ nanorods are distributed evenly on the surface of the CC. EDS mapping images (Figure S20d–f, Supporting Information) confirm the uniform distribution of Mn, O, and Zn elements. The atomic concentration of the elements in Zn_x_MnO_2_ was revealed from EDS spectra in Figure S20g (Supporting Information) in which Mn, O, and Zn are 17.03%, 36.27%, and 0.32%, respectively.

**Figure 7:**
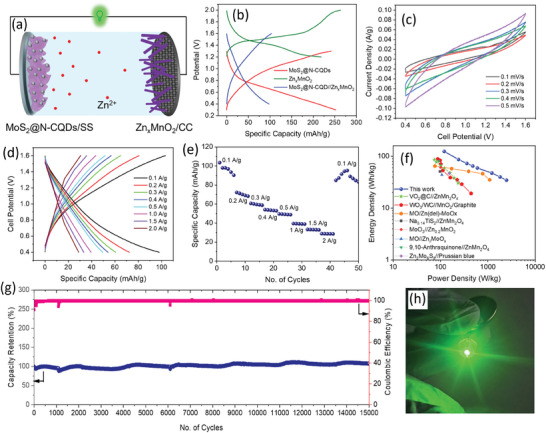
a) Schematic illustration of rocking‐chair MoS_2_@N‐CQDs//Zn_x_MnO_2_ full‐cell ZIB. b) Comparison of GCD profiles of full‐cell battery with those for MoS_2_@N‐CQDs and Zn_x_MnO_2_ in half‐cell configurations at 0.1 A g^−1^. c) CV and d) GCD curves of full‐cell ZIB at various scan rates and current densities, respectively. e) Rate capability and reversibility studies of full‐cell. f) Ragone plot comparing energy‐power densities of MoS_2_@N‐CQDs//Zn_x_MnO_2_ full‐cell with other literature reports, g) cycle performance analyses of the full‐cell battery and h) photograph of the two series connected full‐cells to power a green LED).

Since there is no significant use of Zn in this configuration, significantly increased energy density could be expected in comparison with Zn‐metal batteries. **Figure** **7**a shows the schematic representation of the MoS_2_@N‐CQDs//Zn_x_MnO_2_ full‐cell ZIB. Figure 7b compares the GCD profiles of MoS_2_@N‐CQDs and Zn_x_MnO_2_ in half‐cell configurations and MoS_2_@N‐CQDs//Zn_x_MnO_2_ full‐cell at 0.1 A g^−1^. A high plateau potential of Zn_x_MnO_2_ (≈1.4 V) and a lower discharge slope of MoS_2_@N‐CQDs (within the range of 0.6–0.8 V) suggested a maximum working potential of the full‐cell ZIBs to be ≈1.2 V (between 0.4–1.6 V). Therefore, CVs were recorded within 0.4–1.6 V potential window by varying scan rates from 0.1 to 0.5 mV s^−1^. As depicted in Figure 7c, quasi‐rectangular‐shaped CV curves suggest the contribution of both faradaic and non‐faradaic reactions in the charge storage mechanism.^[^ ^38^ ^]^ Figure 7d depicts the GCD curves of full‐cell measured at varying currents from 0.1 to 2 A g^−1^, in which a reversible discharge specific capacity ≈100 mAh g^−1^ could be achieved at 0.1 A g^−1^. As in Figure 7e, capacity decays with increasing current densities, however, after 40 cycles, a capacity of 85 mAh g^−1^ could be obtained when the current was back at 0.1 A g^−1^, suggesting good reversibility of electrode materials. Interestingly, the specific capacity increased further in successive cycles and reached a maximum value of 95 mAh g^−1^ at the 45th cycle, which might be due to the electrochemical activation, as also observed for MoS_2_@N‐CQDs during half‐cell measurements. Moreover, the green LED was lightened by connecting two full cells in a series (inset of Figure 7e), which signifies their practical applicability.^[^ ^47^, ^48^, ^49^ ^]^ Based on the specific capacity and potential window, the energy/power densities of the MoS_2_@N‐CQDs//Zn_x_MnO_2_ full‐cell ZIBs were calculated at different current densities and compared with the reported literature as depicted in the Ragone plot in Figure 7f. A maximum energy density of 120.3 Wh kg^−1^ was obtained at a power density of 120 W kg^−1^ (calculated from the total active mass of both the active materials), which is higher than most of the recently reported rocking‐chair type ZIBs such as WO_3_/WC//MnO_2_/Graphite,^[^ ^10e^ ^]^ VO_2_@C//ZnMn_2_O_4_,^[^ ^10d^ ^]^ MO//Zn_δ_MoO_x_,^[^ ^50^ ^]^ h‐MoO_3_//Zn_0.2_MnO_2_,^[^ ^10a^ ^]^ Na_0.14_TiS_2_//ZnMn_2_O_4_,^[^ ^11a^ ^]^ 9,10‐Anthraquinone//ZnMn_2_O_4_,^[^ ^13a^ ^]^ Zn‐PB//PTCDI/rGO,^[^ ^13b^ ^]^ and Zn_2_Mo_6_S_8_//Prussian blue.^[^ ^51^ ^]^ Large specific capacities of the cathode and anode materials, along with a high working potential of 1.2 V, resulted in a superior energy density of the system. The proposed cathode and anode reactions during charge/discharge of MoS_2_@N‐CQDs//Zn_x_MnO_2_ full‐cell can be summarized as follows.

Anode:

(5) $$\mathrm{Charging}:\mathrm{xZ}n^{2+}+2xe^{-}+\mathrm{Mo}S_{2}\to Zn_{x}\mathrm{Mo}S_{2}$$

(6) $$\mathrm{Discharging}:Zn_{x}\mathrm{Mo}S_{2}\to \mathrm{xZ}n^{2+}+2xe^{-}+\mathrm{Mo}S_{2}$$

Cathode:

(7) $$\mathrm{Charging}:Zn_{x}\mathrm{Mn}O_{2}-2e^{-}\to Zn_{y}\mathrm{Mn}O_{2}+Zn^{2+}$$

(8) $$\mathrm{Discharging}:Zn_{y}\mathrm{Mn}O_{2}+Zn^{2+}\to Zn_{x}\mathrm{Mn}O_{2}-2e^{-}$$

Furthermore, to explore the practical applicability of the full‐cell batteries, cycling performance was studied for 15 000 cycles at 2 A g^−1^ current density. Notably, the full‐cell demonstrated an ultralong cycle life with almost 97% capacity retention after 15 000 cycles and with almost 100% Coulombic efficiency (Figure 7g). Such ultra‐stable cycling performance of MoS_2_@N‐CQDs//Zn_x_MnO_2_ full‐ell can be attributed to the high cyclic stability of the MoS_2_@N‐CQDs as observed during the half‐cell analyses and rocking‐chair configuration of the full‐cell battery. EIS spectra of the full‐cell were measured before and after cycling studies, as shown in Figure S21 (Supporting Information), where the R_s_ values were found to change minutely from 4.081 to 4.281 ohm before and after 15 000 cycles, respectively, suggesting excellent structural stability of electrode materials. Furthermore, the cycling performance of the MoS_2_@N‐CQDs//Zn_x_MnO_2_ full‐cell at a lower current (0.2 A g^−1^) was also studied, in which 85.8% capacity retention with 98.9% Coulombic efficiency was obtained after 1000 cycles (Figure S22, Supporting Information). It further states that the fabricated full‐cell has shown excellent cycle life in both high current and low current. Minute fluctuations observed in the capacity retention and corresponding Coulombic efficiency might be due to the environmental effects (such as temperature) and poor recording time of the battery testing system (Neware).^[^ ^42^ ^]^ Moreover, the green LED was lightened by connecting two full cells in a series (Figure 7h), which signifies their practical applicability.^[^ ^47^, ^48^, ^49^ ^]^ The high energy density and excellent cyclic stability of the MoS_2_@N‐CQDs//Zn_x_MnO_2_ full‐cell can be explained based on the basis of the following attributes: i) MoS_2_@N‐CQDs provides high Zn^2+^ storage capacity and excellent cyclic stability owing to their rich interlayer dislocations and increased interlayer spacings; ii) Zn_x_ pre‐intercalation provides stable MnO_2_ tunnel structure with high conductive α‐phase and high‐voltage plateau region; iii) benefitted by this, the MoS_2_@N‐CQDs//Zn_x_MnO_2_ cell offered a wide and stable working potential window (1.2 V) which resulted in a high energy density, and finally, iv) absence of Zn‐metal anode mitigates dendrite/corrosion induced capacity fading during ultralong charge/discharge cycles, thus providing excellent cycle stability over thousands of cycles.^[^ ^8^ ^]^

## 3 Conclusion

In summary, N‐CQDs were effectively used to tune the Zn^2+^ ion storage performance of MoS_2_ nanosheets in both half‐cell and full‐cell configurations. The presence of N‐CQDs has induced a large interlayer spacing of 0.8 nm and abundant lattice dislocations in MoS_2_ lattice and also increased its hydrophilicity (water contact angle of 16.78°) for better adsorption, diffusion, and storage of Zn^2+^ ions. For instance, the MoS_2_@N‐CQDs have demonstrated excellent Zn‐ion storage capability with a maximum specific capacity of 258 mAh g^−1^ (at 0.1 A g^−1^), twice that of pristine MoS_2_, and an exceptional capacity retention of 94.5% after 2000 cycles, which is far better than the pristine MoS_2_. Zn‐ion storage kinetic studies revealed that the N‐CQDs induced expanded interlayer spacings in MoS_2_ also elevate Zn^2+^ ions diffusivity, with an excellent diffusion coefficient in the order of 10^−6^ to 10^−8^ cm^2^ s^−1^ being observed, as also supplemented by the mechanistic insights of Zn^2+^ dynamics inside expanded and dislocated MoS_2_ structure. Further, the expanded interlayers could easily accommodate volume variations in MoS_2_ lattice upon Zn‐ion intercalation/deintercalation, as observed through ex‐situ Raman studies, resulting in excellent cycling stability. MoS_2_@N‐CQDs also performed satisfactorily in a zinc‐metal‐free rocking chair ZIB in combination with Zn_x_MnO_2_ as cathode and offered a high energy density and an ultra‐stable cycle life over thousands of cycles. However, the engineering parameters of the battery are yet to be optimized, so further improvement in the device performance is highly likely. Overall, the superior performance of the MoS_2_@N‐CQDs‐based Zn‐metal free battery opens up new possibilities for the widespread applicability of high energy‐dense aqueous zinc ion batteries.

## 4 Experimental Section

### Synthesis of N‐CQDs

Nitrogen‐doped carbon quantum dots (N‐CQDs) were synthesized using a facile solvothermal approach with citric acid (1 g) as the carbon source and urea as the nitrogen (N) source. 1 g of citric acid and 1 g of urea were dissolved in 15 mL of the formamide solvent by constant stirring. Then, the obtained clear solution was heated to 180 °C for 12 h in a 100 mL Teflon‐lined autoclave in a hot air oven. After cooling down the reaction mixture to room temperature, the black‐colored colloidal solution was collected.^[^ ^21b^ ^]^

### Synthesis of MoS_2_@N‐CQDs

For the synthesis of MoS_2_@N‐CQDs, 6 mL of the previously prepared N‐CQDs solution was added to an aqueous solution (72 mL) containing sodium molybdate (3.327 g) and thiourea (6.84 g). The mixture was stirred well in a magnetic stirrer for 1 h, followed by the hydrothermal treatment at 200 °C for 24 h in a 100 mL Teflon‐lined autoclave. The black‐colored precipitate was collected once it cooled down to room temperature, followed by washing with deionized (DI) water and ethanol several times to neutralize the pH. Finally, the MoS_2_@N‐CQDs were obtained by overnight vacuum drying at 80 °C.

### Synthesis of Zn_x_MnO_2_/CC

For the cathode in full‐cell ZIBs, Zn_x_MnO_2_ was prepared by a previously reported method.^[^ ^46^ ^]^ Briefly, KMnO_4_ (1.264 g) and Zn(NO_3_)_2_·6H_2_O (2.379 g) were mixed in DI water (75 mL). Then, 2 mL of H_2_SO_4_ (98%) was added and continuously stirred for 15 min. Along with the pre‐cleaned carbon cloth (CC), the reaction mixture was transferred into a Teflon‐lined autoclave and performed hydrothermal reaction at 140 °C for 3.5 h. Once the reaction medium was cooled down to room temperature, the resultant CC was sonicated for a few seconds to remove the loosely stuck particles. Finally, after drying it in the oven, the Zn_x_MnO_2_/CC was obtained.

### Materials Characterization

The structural properties of MoS_2_@N‐CQDs were investigated through XRD and Raman spectroscopic analyses. XRD was performed through a Panalytical X‐pert PRO diffractometer with Cu‐Kα irradiation within the 2θ range of 5–50°. Airix Corp. STR 500 Raman spectrophotometer with a 15 mW powered 532 nm laser source was used for the Raman analysis. Morphological features of the as‐synthesized pristine and modified MoS_2_ nanostructures were studied by SEM technique using the Nova Nano FE‐SEM‐450 instrument. The functional groups present in the samples were studied through FTIR (Perkin Elmer) between 4000 and 400 cm^−1^ in KBr mode. XPS was performed by ESCA+ Omicron Nano Technology instrument to investigate the elemental and phase configuration of the materials. For revealing the detailed morphological, structural, and compositional features, TEM was performed using a TALOS F200S G2 instrument with SAED, STEM and EELS facilities. Contact angle measurements were performed through the Sessile drop method using the KYOWA interface measurement and analysis system. Thermal stability and the amount of MoS_2_ in MoS_2_@N‐CQDs were investigated using TGA (STA 2500 thermogravimetric analyzer). Moreover, the optical properties of the synthesized N‐CQDs were analyzed through a UV–visible spectrophotometer (Agilent) and fluorescence spectrometer (LS 55, Perkin Elmer).

### Electrode Fabrication and Cell Assembly

For half‐cell configuration, the active material (MoS_2_ or MoS_2_@N‐CQDs) was used as a cathode and Zn‐foil was used as the counter/reference electrode. For electrode preparation, the active material was mixed with acetylene black and polyvinylidene fluoride (PVDF) in the weight ratio of 8:1:1 and grounded well in a mortar and pestle. N‐methyl‐2‐pyrrolidone was added in a few drops to make a homogeneous slurry of the materials. Then, it was coated in stainless steel (SS) circular electrodes having a diameter of 14 mm using doctor blades and kept in the oven for 8 h at 80 °C for drying. The mass of the fabricated electrodes is in the range of 0.7 to 1.2 mg. The cell was assembled in CR2032 coin‐cells by keeping 3 m Zinc trifluoromethanesulfonate (Zn(OTf)_2_) electrolyte‐soaked Whatman filter paper (separator) in between Zn‐foil (anode) and as‐prepared electrode (cathode). Finally, after crimping the coin cell, it was kept constant for 24 h for better electrolyte diffusion into the electrodes.

For rocking‐chair Zn metal‐free full‐cell configuration, MoS_2_@N‐CQDs and Zn_x_MnO_2_/CC were used as an anode and cathode, respectively, and assembled in CR2032 coin‐cells by following the same procedure as of half‐cell. The mass ratio of the anode and cathode was taken as 1:1, and all the calculations were performed by considering the total active mass of both electrodes.

### Electrochemical Characterizations

For electrochemical performance analysis, CV, GCD, and EIS were used for both half‐cell and full‐cells. Within the working potential window (0.3–1.3 V for half‐cell and 0.4–1.6 V for full‐cell), CV was performed at different scan rates, and GCD was performed at different current densities. EIS was performed in OCP with the initial perturbation potential of 5 mV between 100 kHz and 10 mHz frequency range. GITT was also performed with 8 min of (dis)charging at 0.1 A g^−1^ and 32 min of rest. Characterizations such as CV and EIS were performed using the CHI760E workstation, while GCD and GITT were measured in the Neware BTS4000‐5V20 mA battery testing system.

### Molecular Dynamic Simulations

**Figure 8:**
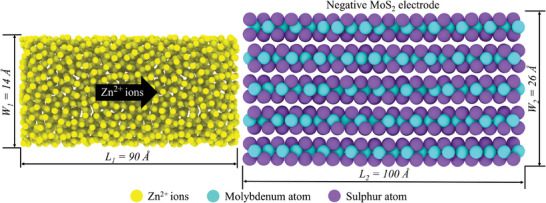
Snapshot of the simulation box packed with MoS_2_ (right) and Zn‐ions (left). The Zn‐ions are colored yellow and packed along with MoS_2._

This study employed MD simulations to comprehend the Zn‐ion storage in the MoS_2_ electrodes (pristine and N‐CQDs dislocated MoS_2_). Charmm GUI was employed to develop the five‐layer parallel plate MoS_2_ electrode structures for the simulation.^[^ ^52^ ^]^ Later, the MoS_2_ structure was dislocated in the middle layer (3rd layer) to develop the N‐CQDs‐based MoS_2_ electrodes. Then, these two electrodes were separately packed in two different simulation boxes with a dimension of 5.0 nm × 10.0 nm × 2.6 nm, each with periodic boundary conditions. After developing, they are packed with Zn‐ions in a simulation box of 5.0 nm × 19.2 nm × 2.6 nm with periodic boundary conditions. After packing, the constant charge method was employed to get insights into corresponding Zn‐ion storage inside the two MoS_2_ electrodes. Charmmfsw interatomic potential was used to define the rule governing the interactions between the atoms in the system.^[^ ^53^ ^]^ **Figure** **8** shows five layered MoS_2_ electrodes packed with Zn‐ions. Packmol software was used to pack the simulation box containing the MoS_2_ electrode and Zn‐ions.^[^ ^54^ ^]^ All computations were performed with a timestep of 0.1 fs using LAMMPS (large‐scale atomic/molecular massively parallel simulator) software.^[^ ^55^ ^]^ The results were post‐processed using OVITO PRO software.^[^ ^56^ ^]^

## Conflict of Interest

The authors declare no conflict of interest.

## Supporting information



Supporting Information

## Data Availability

The data that support the findings of this study are available from the corresponding author upon reasonable request.
