# Supplementary material for: Insights into Interlayer Dislocation Augmented Zinc‐Ion Storage Kinetics in MoS2 Nanosheets for Rocking‐Chair Zinc‐Ion Batteries with Ultralong Cycle‐Life
Source: Small. 2025 Jan 9;21(6):2410408. doi: 10.1002/smll.202410408 (PMC11817946; doi:10.1002/smll.202410408)
Supplement: Supplementary file 1 — Supporting Information [file SMLL-21-2410408-s001.pdf]

NANO · MICRO
small

Supporting Information

for *Small*, DOI 10.1002/smll.202410408

Insights into Interlayer Dislocation Augmented Zinc-Ion Storage Kinetics in MoS_2 Nanosheets for Rocking-Chair Zinc-Ion Batteries with Ultralong Cycle-Life

Muruganandham Hariram, Pankaj K. Pal, Anusree S. Chandran, Manikantan R. Nair, Manoj Kumar, Mukhesh K. Ganesha, Ashutosh K. Singh, Basundhara Dasgupta, Saurav Goel, Tribeni Roy, Prashanth W. Menezes and Debasish Sarkar**

Supporting Information

Insights into Interlayer Dislocation Augmented Zinc-ion Storage Kinetics in MoS_2 Nanosheets for Rocking-Chair Zinc-Ion Batteries with Ultralong Cycle-life

Muruganandham Hariram, Pankaj K. Pal, Anusree S. Chandran, Manikantan R. Nair, Manoj Kumar, Mukhesh K. Ganesha, Ashutosh K. Singh, Basundhara Dasgupta, Saurav Goel, Tribeni Roy, Prashanth W. Menezes, Debasish Sarkar**

M. Hariram, P. K. Pal, M. Kumar, D. Sarkar
Department of Physics, Malaviya National Institute of Technology Jaipur, Rajasthan 302017, India. E-mail: debasish.phy@mnit.ac.in

A. S. Chandran, M. R. Nair, T. Roy
Department of Mechanical Engineering, Birla Institute of Technology and Science, Pilani (BITS Pilani), Rajasthan 333031, India

M. K. Ganesha, A. K Singh
Centre for Nano and Soft Matter Sciences, Bengaluru 562162, India

B. Dasgupta, P. W. Menezes
Department of Chemistry, Technical University of Berlin, Straße des 17 Juni 135. Sekr. C2, 10623, Berlin, Germany. E-mail: prashanth.menezes@mailbox.tu-berlin.de

S. Goel
School of Engineering, London South Bank University, London, SE1 0 AA, UK

S. Goel
University of Petroleum and Energy Studies, Dehradun 248007, India

P. W. Menezes
Material Chemistry Group for Thin Film Catalysis – CatLab, Helmholtz-Zentrum Berlin für Materialien und Energie, Albert-Einstein-Str. 15, 12489 Berlin, Germany. E-mail: prashanth.menezes@helmholtz-berlin.de

P. W. Menezes
University of Southern Queensland, Centre for Future Materials (CFM), QLD, 4350, Australia

Calculation details

(i) Calculation of the concentration of MoS_2 in MoS_2@N-CQDs from TGA:

The concentration of MoS_2 in MoS_2@N-CQDs is calculated from the following reaction:

$$MoS_2\ (wt\%) = 100 \times \frac{M_{MoS_2}}{M_{MoO_3}} \times \frac{Final\ weight\ of\ MoO_3}{Initial\ weight\ of\ MoS_2@N{-}CQDs} \quad \text{(S1)}$$

Here. M represents molecular weight.

ii) Amount of Zn^{2+} ions de(intercalated) in the electrode materials:

The amount of Zn^{2+} ions (*x*Zn) (de)intercalated into the electroactive material is calculated from Faraday's equation,

$$Q_{\text{theoretical}} = \frac{2nF}{3600 \times M} \quad \text{(S2)}$$

where $Q_{\text{theoretical}}$ is the theoretical capacity of MoS_2 (mAh/g), n is the number of Zn-ions accommodated per unit formula (n=2), F is the Faraday constant (96,485 C/mol), and M is the molecular weight of MoS_2. By calculating the ratio of $Q_{\text{theoretical}}$ to $Q_{\text{experimental}}$, the *x* value can be obtained.

(iii) Segregation of capacitive and diffusive parts from CV:

To evaluate the charge storage mechanism of ZIBs, current (*i*) and scan rate (*v*) were analyzed from the CV curve by using the following reaction:

$$i = av^b \quad \text{(S3)}$$

Here a and b are variable parameters. The value of b can be calculated by the slope of log *i* versus log *v* plot, which provides the charge storage kinetics. If the value of *b* is 1, the capacitive process is dominating while the diffusive process is dominating when *b* is 0.5. For segregating the capacitive and diffusive currents, the following formula is used.

$$i(V) = a_1 v + a_2 v^{\frac{1}{2}} \quad \text{(S4)}$$

Here i and v are the current and scan rates. The values of a_1 and a_2 are calculated by finding the slope and intercept of the $i(V)/v^{1/2}$ versus the $v^{1/2}$ plot.

(iv) Calculation of the real part of capacitance:

The real part of the capacitance over the frequency range for the 2D Bode plot was calculated through the following equation,

$$C' = \frac{Z'}{2\pi f |Z|^2} \tag{S5}$$

Where f is the frequency (Hz), $|Z|$ is the absolute impedance (Ω) and Z' is the imaginary part of the impedance (Ω).

(v) Calculation of zinc-ions diffusion co-efficient from GITT:

For GITT studies, the cell was charged/discharged for 8 mins and rested for 32 mins. The diffusion coefficient of Zn-ions (D_{Zn}) was calculated by the following equation:

$$D_{Zn} = \frac{4}{\pi}\left(\frac{m_B V_M}{M_B A}\right)^2 \left(\frac{\mathbf{\Delta E}_S}{\mathbf{\Delta E}_\tau}\right)^2 \tag{S6}$$

Here, τ is the time of charging/discharging (s), m_B is the active mass loading (g), V_M is the molar volume of MoS_2 (cm^3/mol), M_B is the molecular weight of MoS_2 (g/mol) and A is the electrode/electrolyte contact area (cm^2). Moreover, ΔE_τ and ΔE_S are the variations of cell voltage and steady-state voltage, respectively.

(vi) Calculation of Zn-ions concentration:

Unit cell volume of MoS_2 was determined by "a (3.16 Å) × b (3.16 Å) × c (12.29 Å) × sin120° = 106.28 × 10^{-24} cm^3". 1 cm^3 of MoS_2 have 1/106.28 × 10^{-24} = 9.41 × 10^{21} unit cells. In our case, ZIB shows the maximum specific capacity of 258 mAh/g, which signifies 0.67 Zn^{2+} insertion in MoS_2 ($Zn_{0.59}MoS_2$). Each molecule of MoS_2 has 0.59 Zn ions and each unit cell contains 2 molecules. Therefore, 1 cm^3 has (9.41 × 1021/6.02 × 1023) × 2 × 0.59 = 1.84 × 10^{-2} mol Zn-ions.

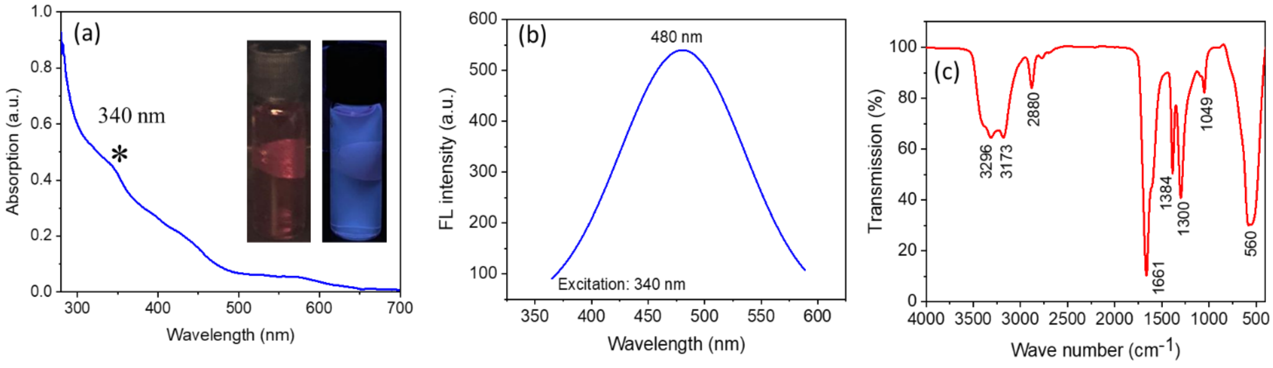

Figure S1. (a) UV-visible absorption spectrum, (b) fluorescence spectrum and (c) FTIR spectrum of N-CQDs (inset of S1 (a): photographs of N-CQDs without and with UV irradiation).

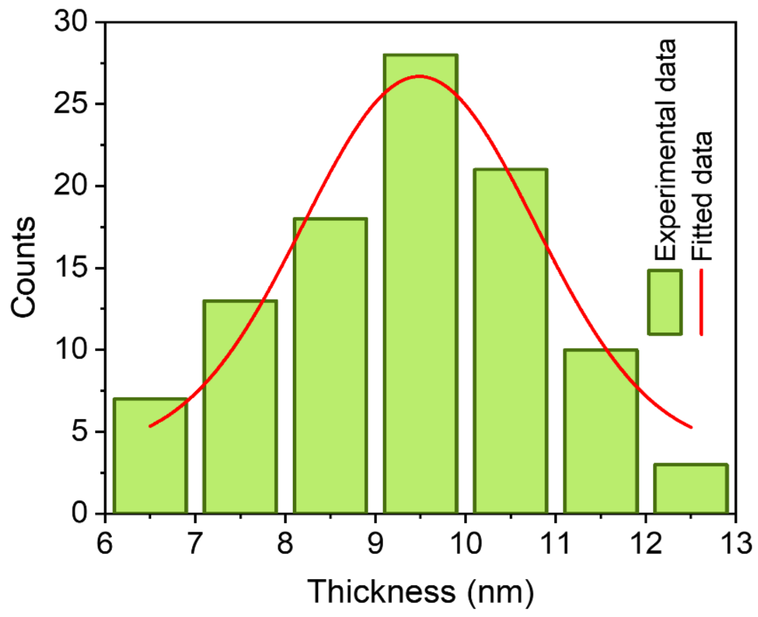

Figure S2. Thickness distribution plot of pristine MoS_2.

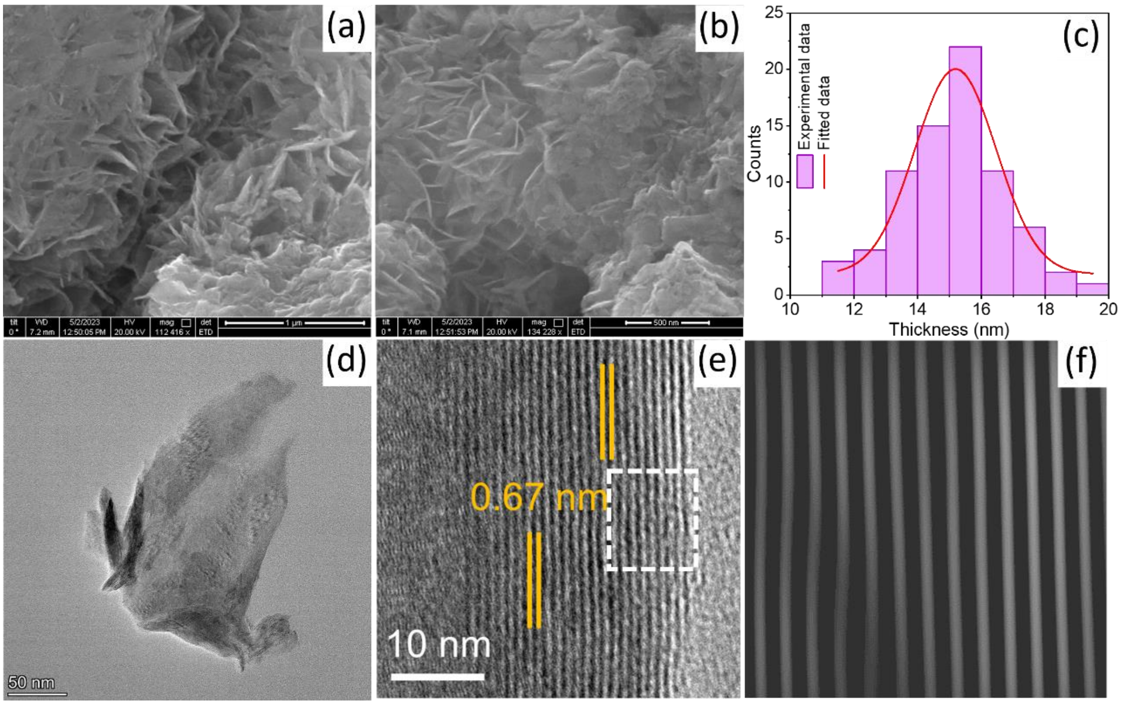

Figure S3. (a-c) SEM image with the corresponding thickness distribution graph, (d) TEM, (e) HRTEM images pristine MoS_2, (f) iFFT image of the white-colored dotted region of (e).

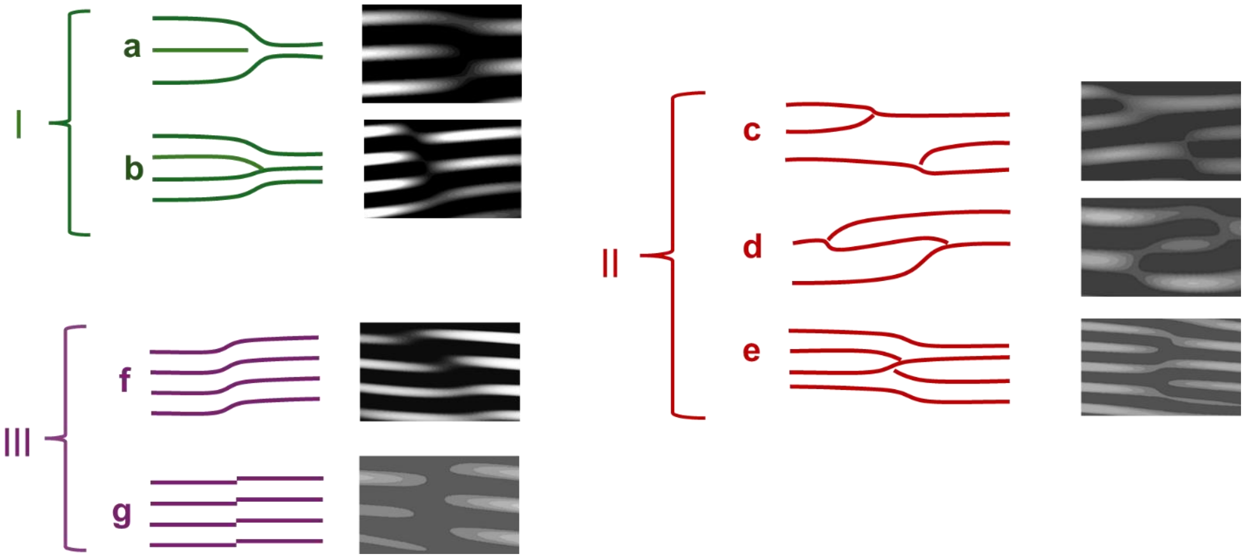

Figure S4. Classification of interlayer dislocations in the MoS_2@N-CQDs with the schematics and the corresponding inverse Fourier-transformed HRTEM images.

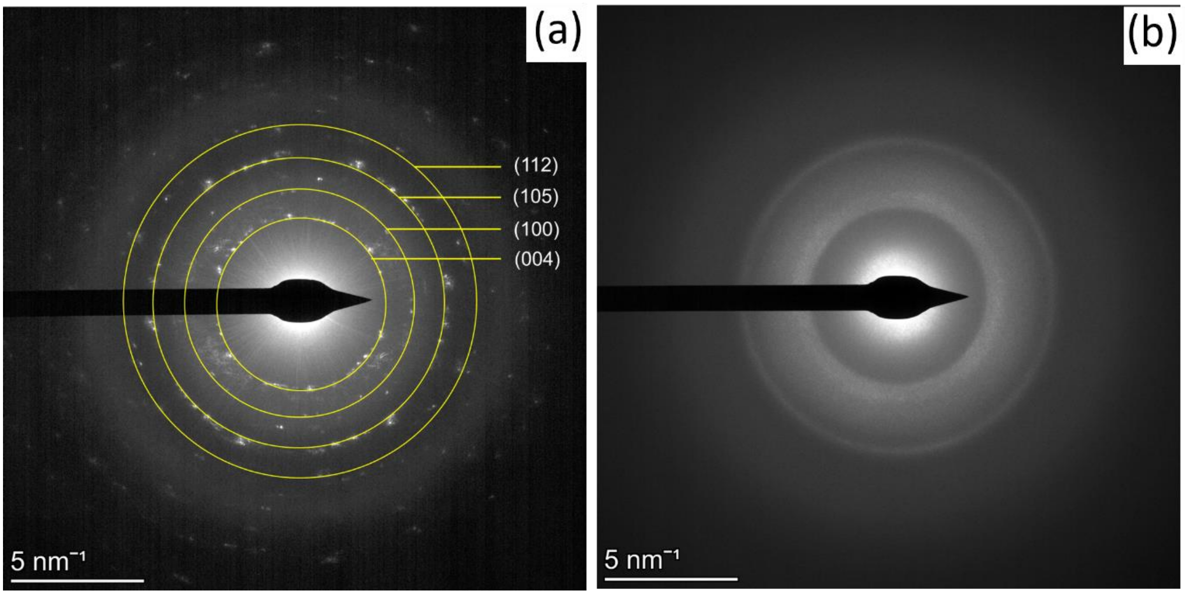

Figure S5. SAED pattern of (a) MoS_2 and (b) MoS_2@N-CQDs

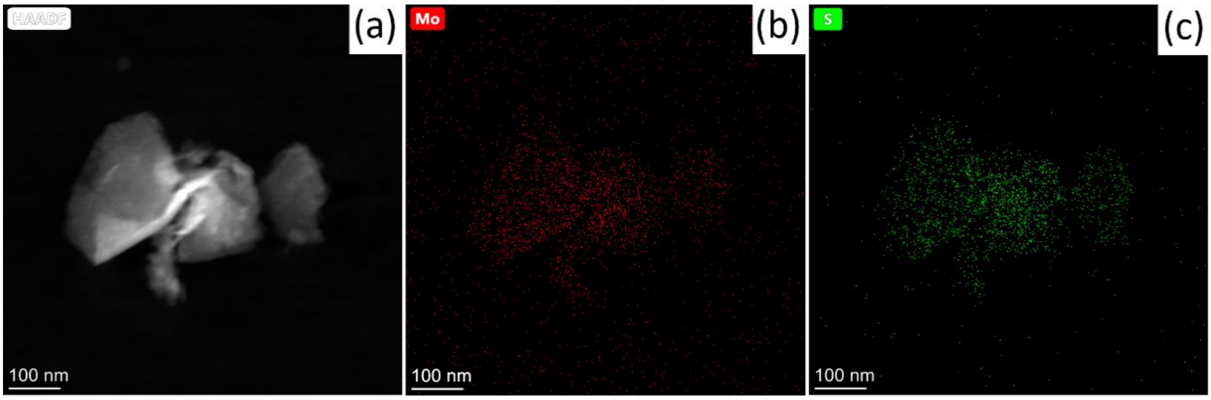

Figure S6. (a) HAADF-STEM image with corresponding EELS mapping for (b) Mo and (c) S elements of MoS_2.

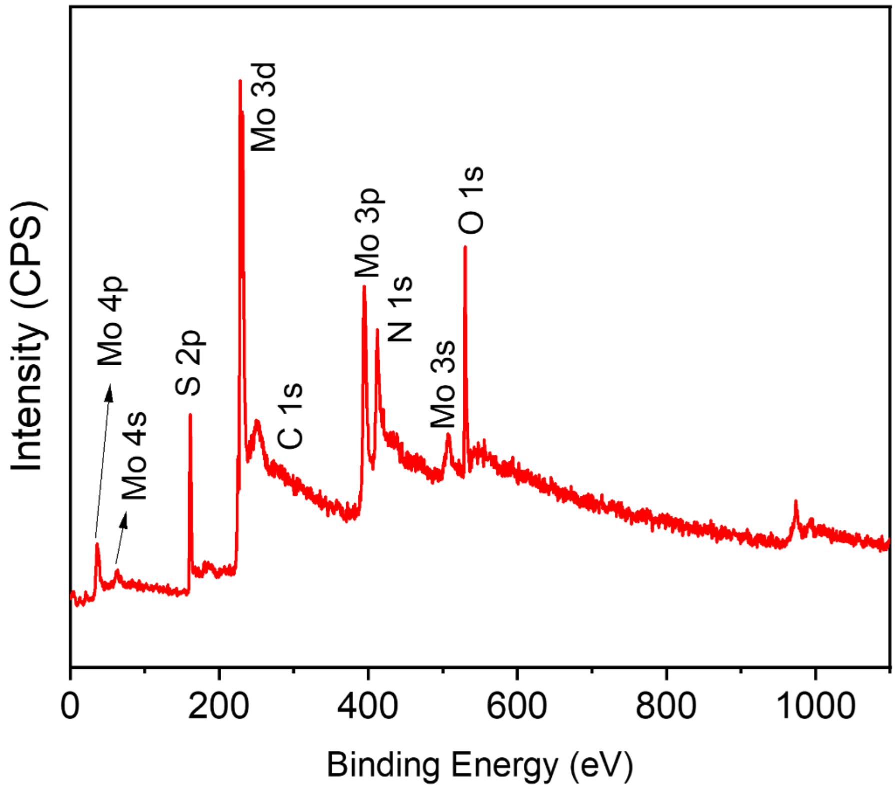

Figure S7. (a) XPS survey spectrum of MoS_2@N-CQDs.

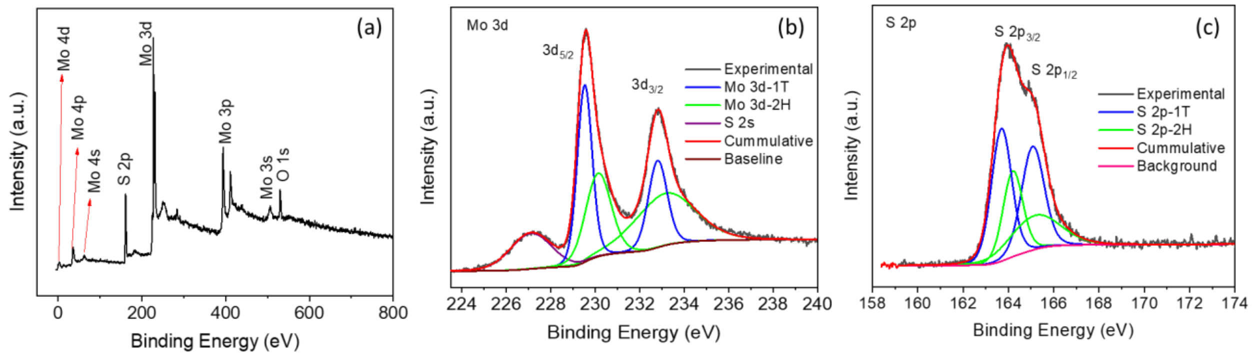

Figure S8. (a) XPS survey spectrum of pristine MoS_2 and (b) its deconvoluted high-resolution XPS spectrum of Mo 3d and S 2p elements.

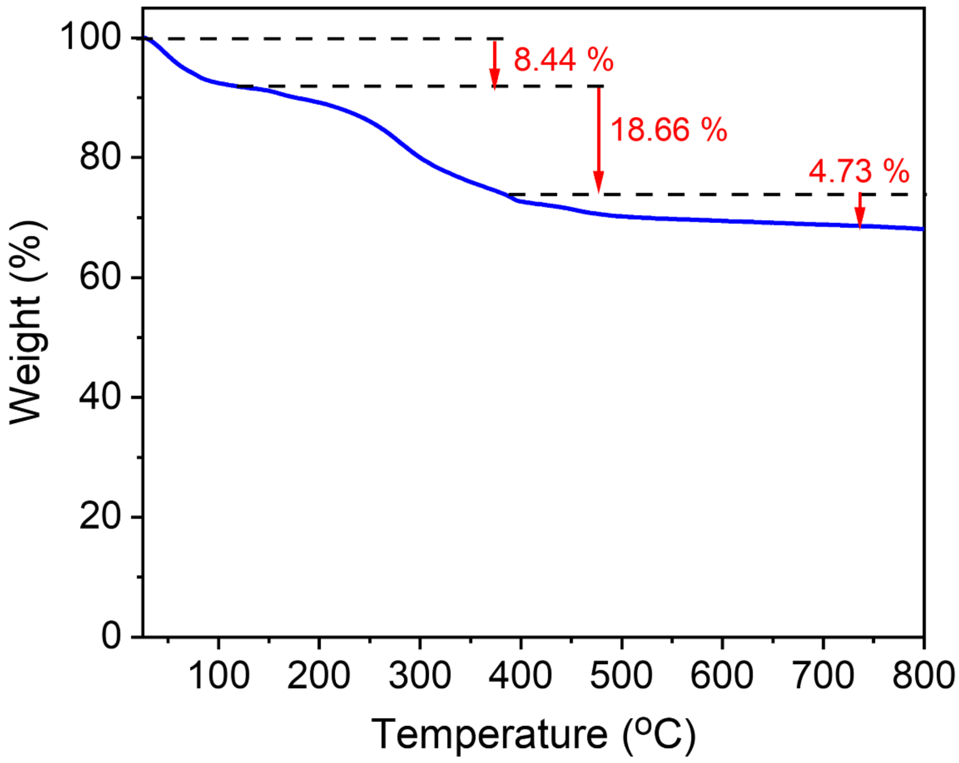

Figure S9. Thermogravimetric analysis of MoS_2@N-CQDs.

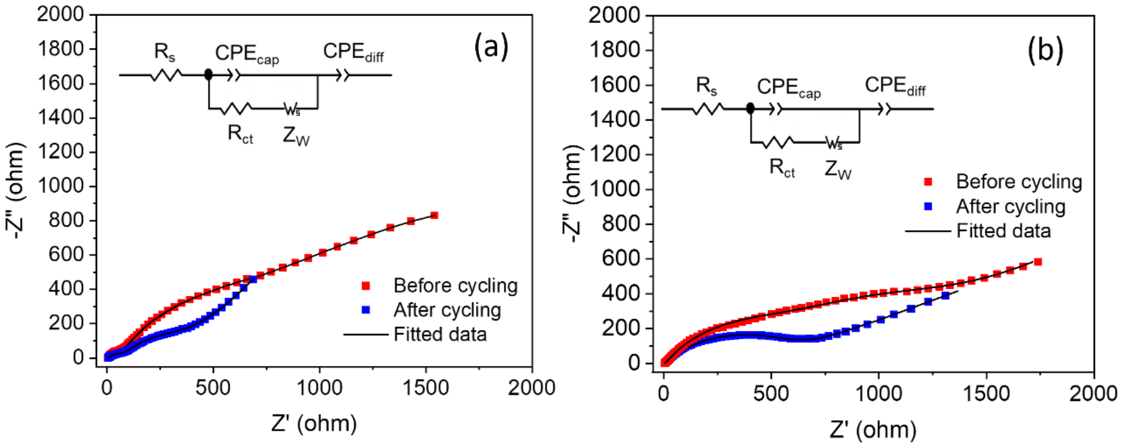

Figure S10. Nyquist plot of (a) MoS_2@N-CQDs and (b) pristine MoS_2 before and after cycle (inset: corresponding equivalent circuit model)

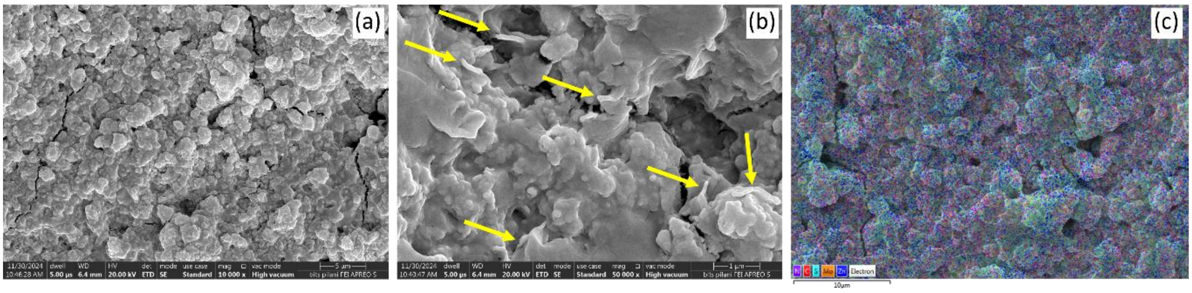

Figure S11. (a, b) SEM and (c) EDS mapping images of MoS_2@N-CQDs after 2000 charge/discharge cycles

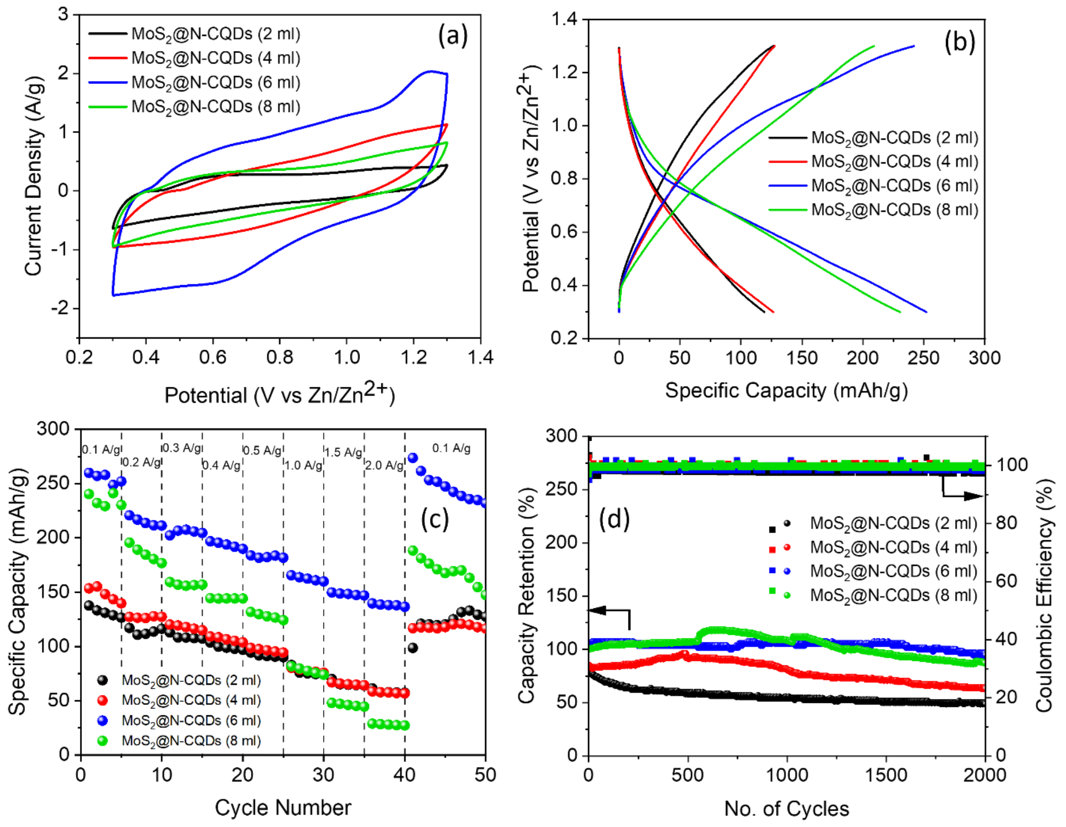

Figure S12. Comparison of (a) CV (at 2 mV/s), (b) GCD (at 0.1 A/g), (c) rate performance, and (d) cyclic stability (at 2 A/g) for MoS_2@N-CQDs with different concentrations of N-CQDs.

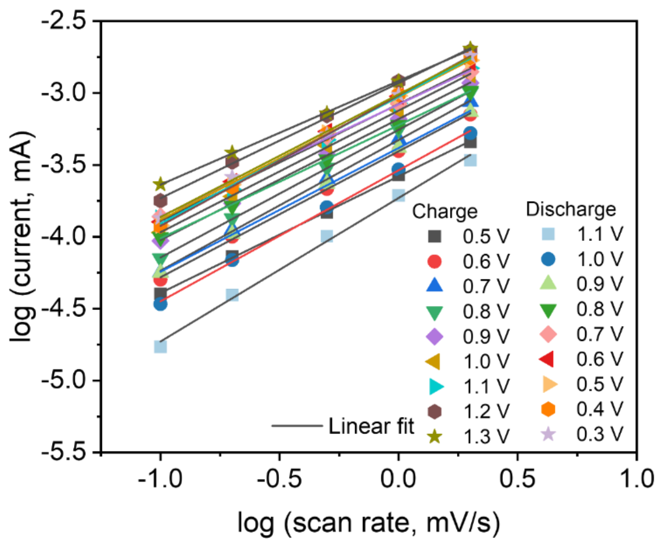

Figure S13. Log (current) vs log (scan rate) plot of MoS_2@N-CQDs.

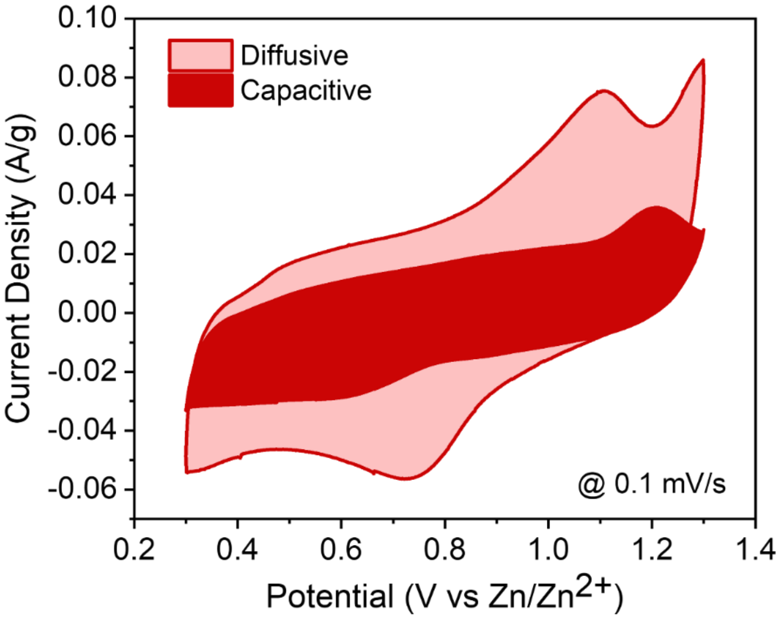

Figure S14. Segregation of capacitive and diffusive Zn^{2+} storage contributions at 0.1 mV/s of MoS_2.

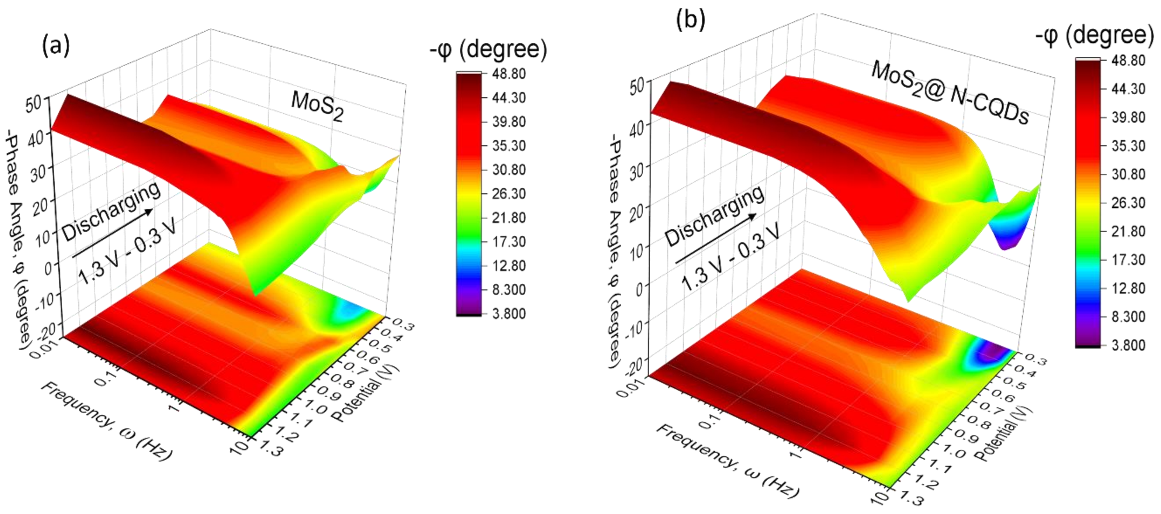

Figure S15. 3D Bode impedance plots of (e) MoS_2 and (f) MoS_2@N-CQDs during discharging

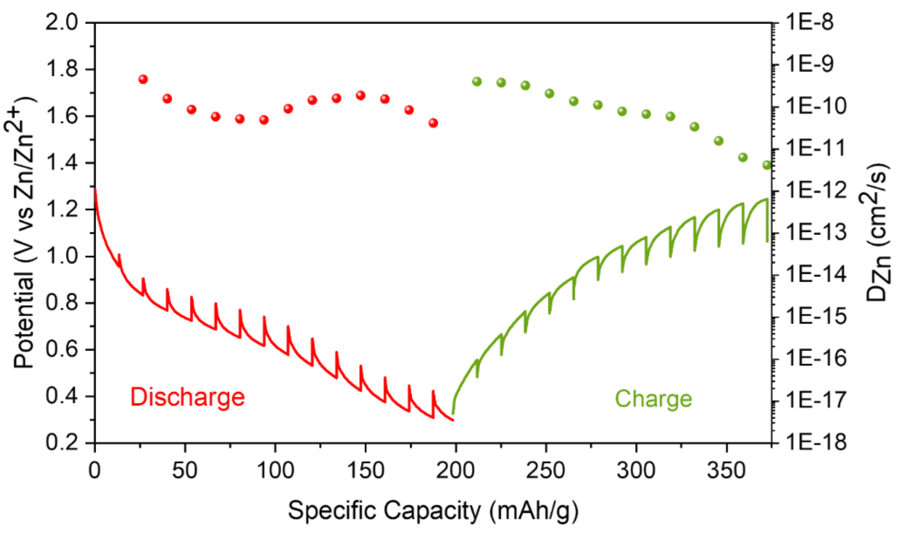

Figure S16. GITT of pristine MoS_2

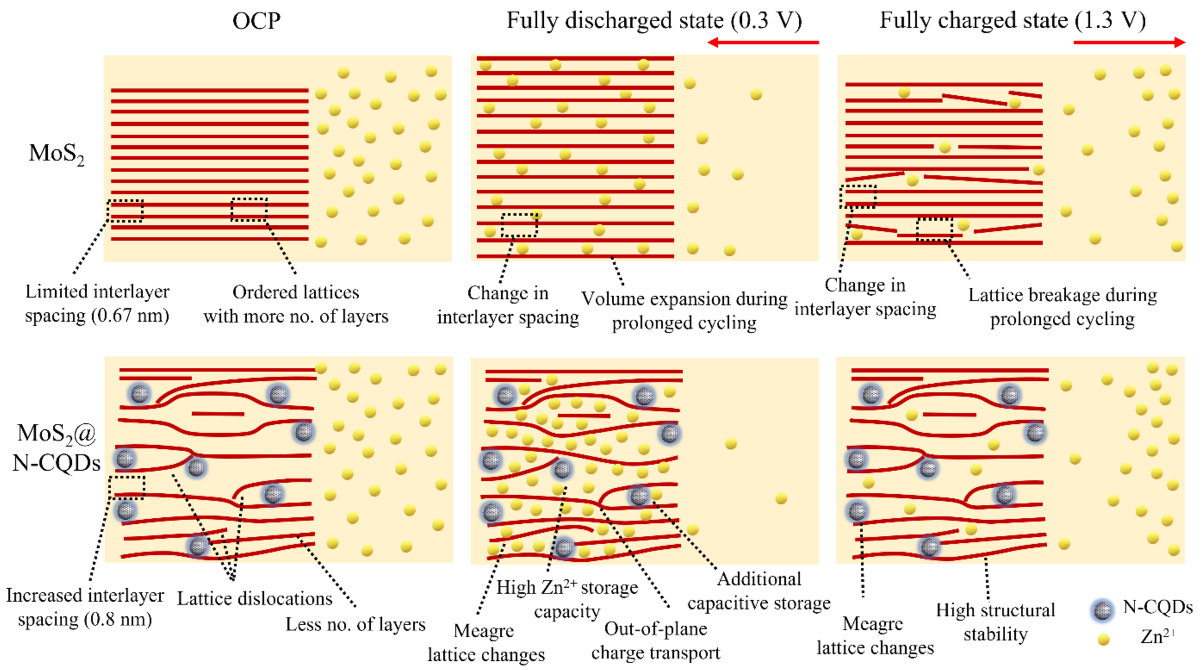

Figure S17. Schematic illustration of Zn-ion storage mechanism in MoS_2 and MoS_2@N-CQDs.

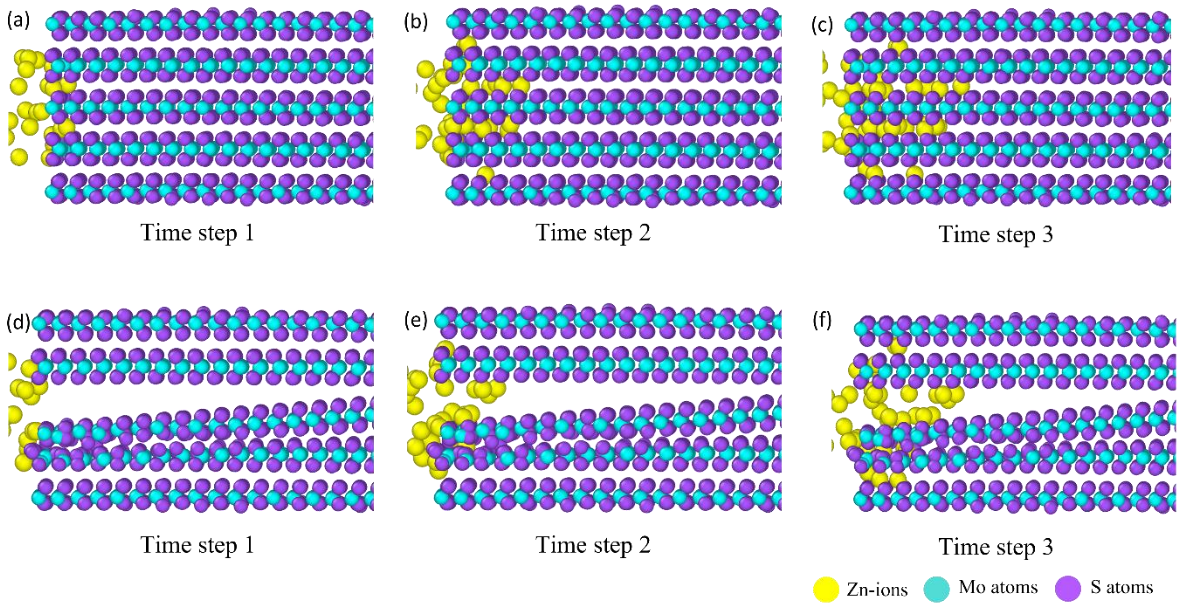

Figure S18. Snapshot of Zn ion storage inside (a-c) ordered MoS_2 and (d-f) dislocated MoS_2 electrodes while applying a low current density at different time steps.

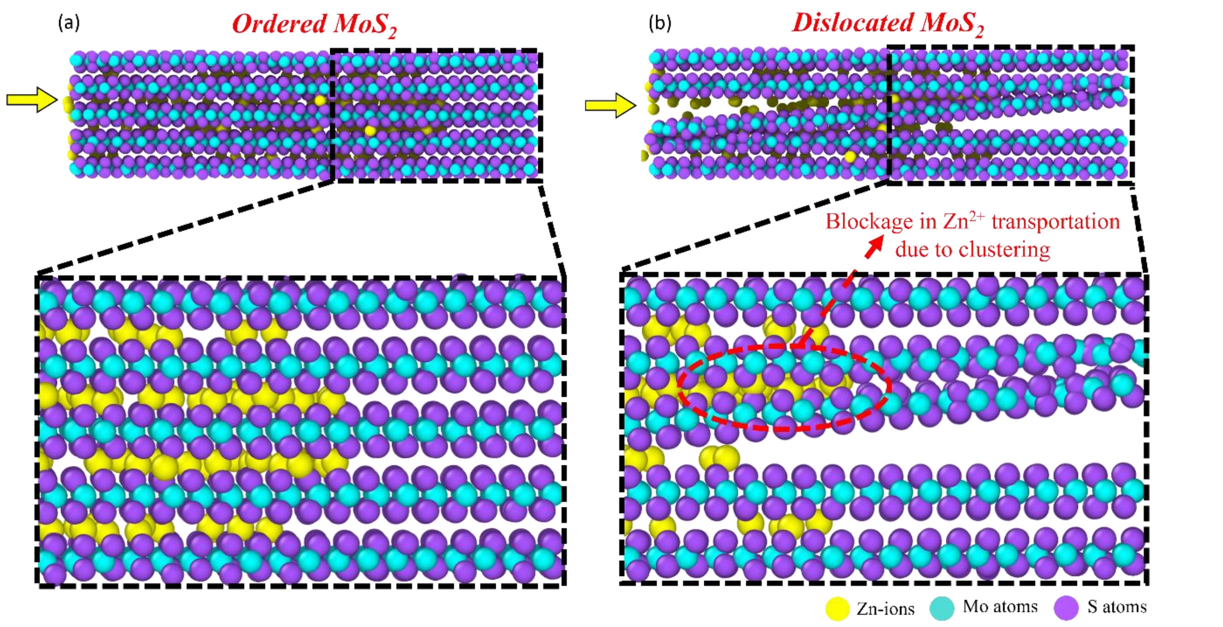

Figure S19. Snapshot of Zn ion storage inside (a) ordered MoS_2 and (b) dislocated MoS_2 electrodes at high current density.

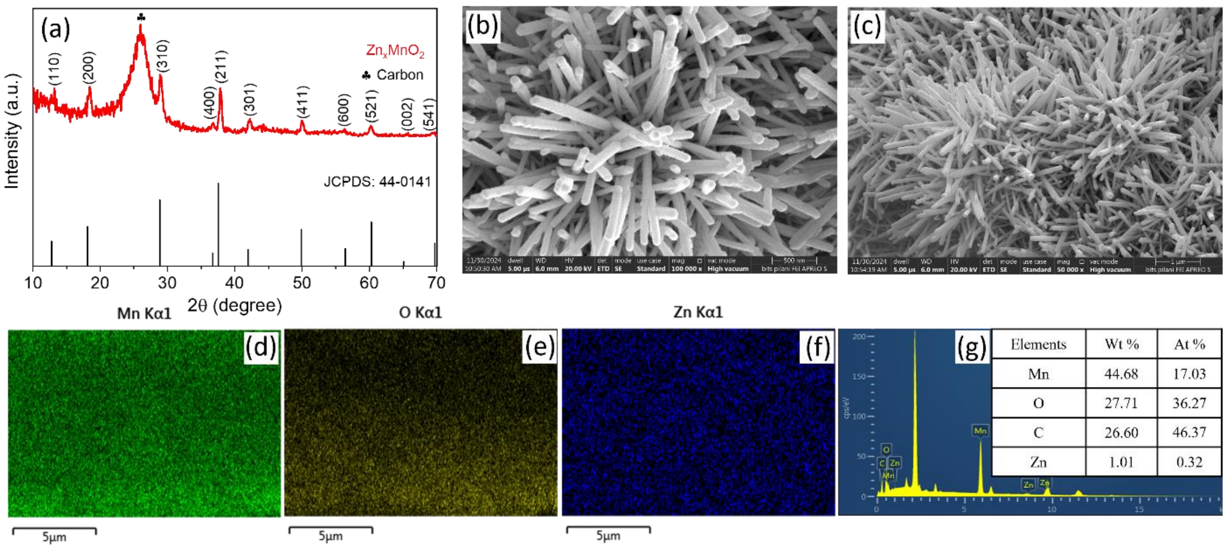

Figure S20. (a) XRD pattern, (b, c) FESEM images, EDS mapping of (d) Mn, (e) O, and (f) Zn elements, and (c) EDS spectrum of Zn_xMnO_2.

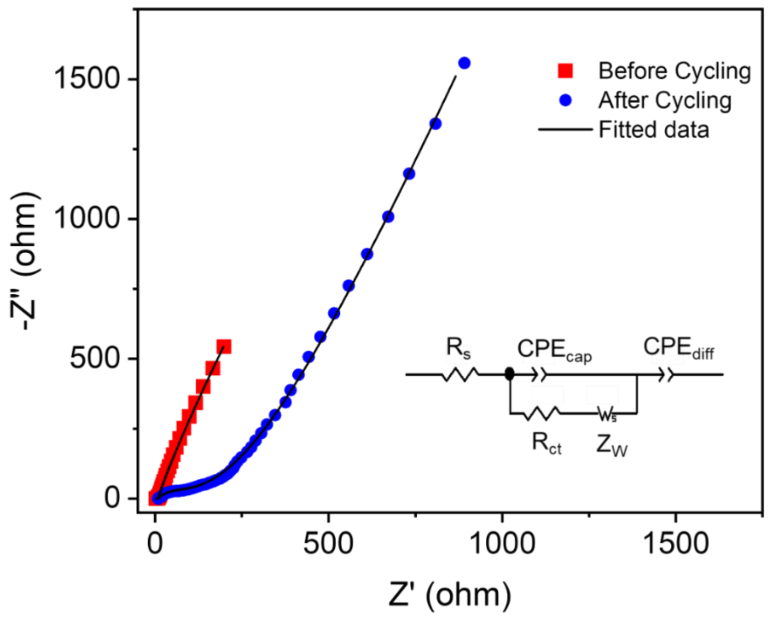

Figure S21. Nyquist plot of MoS_2@N-CQDs//Zn_xMnO_2 battery (inset: equivalent circuit).

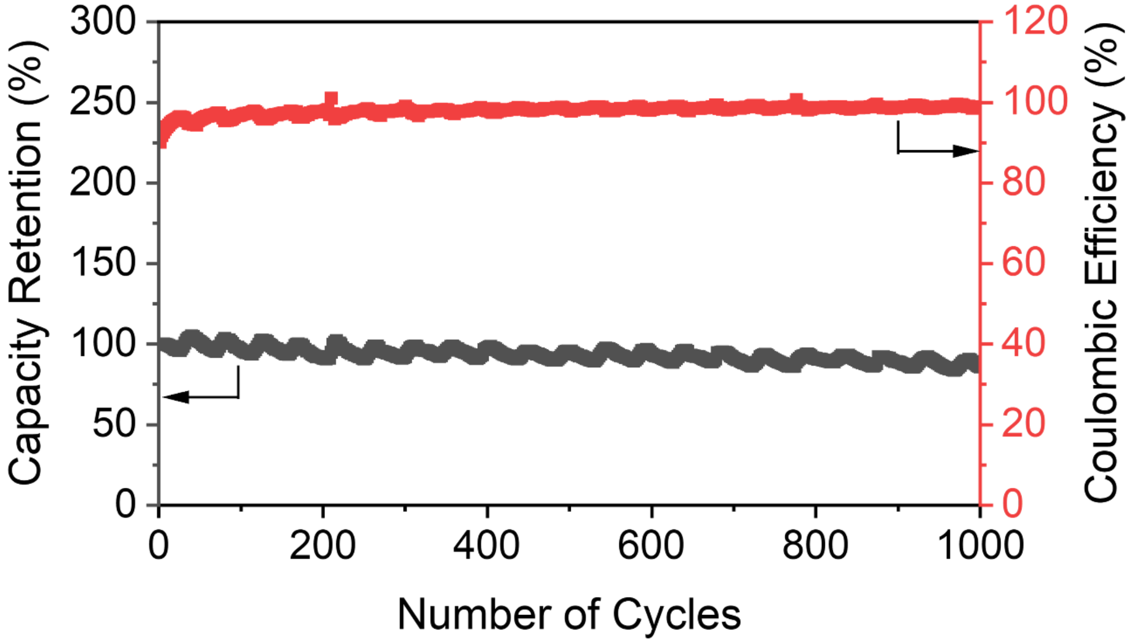

Figure S22. Cyclic performance of MoS_2@N-CQDs//Zn_xMnO_2 battery at 0.2 A/g.

Table S1. Comparison of MoS_2-based cathode materials for ZIBs in half-cell.

Sl. No.	Cathode Material	Interlayer Spacing (nm)	Electrolyte	Voltage (V)	Specific Capacity (@ current density)	Diffusion co-efficient (cm^2/s)	Cyclic Retention (@ no. of cycles)	Ref.
1	MoS_2	-	2 M $ZnSO_4$	0.1-2	18 mAh/g (0.05 A/g)	-	-	[1]
2	MoS_2	0.73	2 M $ZnSO_4$	0.3-1.5	202.6 mAh/g (0.1 A/g)	-	98.6% (600 cycles)	[2]
3	O-MoS_2	0.95	3 M $Zn(CF_3SO_3)_2$	0.2-1.4	232 mAh/g (0.1 A/g)	10^{-8} to 10^{-9}	68% (2000 cycles)	[3]
4	MoS_2 (~70% 1T)	-	3 M $Zn(CF_3SO_3)_2$	0.25–1.25	168 mAh/g (0.1 A/g)	-	98.1% (400 cycles)	[4]
5	MoS_{2-x} (S-vacancy)	0.686	3 M $Zn(CF_3SO_3)_2$	0.25–1.25	138.6 mAh/g (0.1 A/g)	-	87.8% (1000 cycles)	[5]
6	N-MoS_2	0.86	3 M $Zn(CF_3SO_3)_2$	0.2-1.3	149.6 mAh/g (0.1 A/g)	-	89.1% (1000 cycles)	[6]
7	1T MoS_2@CC	0.67	3 M $Zn(CF_3SO_3)_2$	0.25–1.25	198 mAh/g (0.1 A/g)	-	87.8% (2000 cycles)	[7]
8	MoS_2/CTAB	1	3 M $ZnSO_4$	0.2-1.3	181.8 mA/g (0.1 A/g)	10^{-9} to 10^{-12}	~92.8% (2100 cycles)	[8]
9	MoS_2-H_2O	0.91	3 M $Zn(CF_3SO_3)_2$	0.25-1.25	164.1 mAh/g (0.1 A/g)	10^{-11} to 10^{-13}	83.1% (100 cycles)	[9]
10	MWCNTs @amorphous carbon@MoS_2	0.88	3 M PVA-$Zn(CF_3SO_3)_2$	0.13-1.2	181 mAh/g (0.1 A/g)	10^{-10} to 10^{-11}	78% (1000 cycles)	[10]
11	Crystal water intercalated MoS_2	0.78	2 M $ZnSO_4$	0.3-1.3	197 mAh/g (0.1 A/g)	10^{-10} to 10^{-13}	55% (1000 cycles)	[11]

12	Tetramethylammonium/MoS_2	1.06	3 M $Zn(CF_3SO_3)_2$	0.2-1.3	212.4 mAh/g (0.1 A/g)	10^{-10} to 10^{-11}	96.1% (400 cycles)	[12]
13	Rag-like MoS2	0.688	3 M $Zn(CF_3SO_3)_2$	0.2-1.4	232.6 mAh/g (0.1 A/g)	10^{-10} to 10^{-11}	83.8% (2100 cycles)	[13]
14	Se-doped MoS_2	0.65	3 M $Zn(CF_3SO_3)_2$	0.2-1.3	213 mAh/g (0.1 A/g)	10^{-10} to 10^{-11}	74.1% (100 cycles)	[14]
15	MoS_2-double-layer nanotubes	0.71	3 M $Zn(CF_3SO_3)_2$	0.1-1.2	242 mAh/g (0.1 A/g)	10^{-10} to 10^{-11}	75.6 (7000 cycles)	[15]
16	MoS_2-β-cyclodextrin	0.96	2 M $ZnSO_4$	0.25-1.4	228 mAh/g (4 mA/cm^2)	10^{-8} to 10^{-9}	85.3 (5000 cycles)	[16]
17	Mn-MoS_2/MXene	0.91	3 M $Zn(CF_3SO_3)_2$	0.25-1.25	191 mAh/g (0.1 A/g)	10^{-10} to 10^{-12}	80.3 (500 cycles)	[17]
18	Carbon/MoS_x	0.62	2 M $ZnSO_4$	0.2-1.2	125 mAh/g (0.1 A/g)	10^{-10}	88.3 (400 cycles)	[18]
19	MoS_2@ethylene glycol	0.95	3 M $Zn(CF_3SO_3)_2$	0.2-1.25	166.2 mAh/g (0.1 A/g)	-	87.9 (1000 cycles)	[19]
20	MoS_2	0.67	3 M $Zn(CF_3SO_3)_2$	0.3-1.3	116 mAh/g (0.1 A/g)	10^{-9} to 10^{-12}	45 % (2000 cycles)	This work
21	MoS_2@N-CQDs	0.8	3 M $Zn(CF_3SO_3)_2$	0.3-1.3	258 mAh/g (0.1 A/g)	10^{-6} to 10^{-8}	94.5 % (2000 cycles)	This work

Table S2. Comparison of resistive elements of MoS_2 and MoS_2@N-CQDs before and after cycling

Material	Before Cycling (ohm)		After Cycling (ohm)	
	**R_s**	**R_{ct}**	**R_s**	**R_{ct}**
MoS_2@N-CQDs	2.77	4.1	5.66	7.5
MoS_2	4.73	495.5	6.24	690
MoS_2@N-CQDs//Zn_xMnO_2	4.08	4.28	9.46	38.31

Reference

[1] W. Liu, J. Hao, C. Xu, J. Mou, L. Dong, F. Jiang, Z. Kang, J. Wu, B. Jiang, F. Kang, *Chem. Commun.* **2017**, *53*, 6872.

[2] H. Li, Q. Yang, F. Mo, G. Liang, Z. Liu, Z. Tang, L. Ma, J. Liu, Z. Shi, C. Zhi, *Energy Storage Mater.* **2019**, *19*, 94.

[3] H. Liang, Z. Cao, F. Ming, W. Zhang, D. H. Anjum, Y. Cui, L. Cavallo, H. N. Alshareef, *Nano Lett.* **2019**, *19*, 3199.

[4] J. Liu, P. Xu, J. Liang, H. Liu, W. Peng, Y. Li, F. Zhang, X. Fan, *Chem. Eng. J.* **2020**, *389*, 124405.

[5] W. Xu, C. Sun, K. Zhao, X. Cheng, S. Rawal, Y. Xu, Y. Wang, *Energy Storage Mater.* **2019**, *16*, 527.

[6] Z. Sheng, P. Qi, Y. Lu, G. Liu, M. Chen, X. Gan, Y. Qin, K. Hao, Y. Tang, *ACS Appl. Mater. Interfaces* **2021**, *13*, 34495.

[7] J. Liu, N. Gong, W. Peng, Y. Li, F. Zhang, X. Fan, *Chem. Eng. J.* **2022**, *428*, 130981.

[8] Z. Yao, W. Zhang, X. Ren, Y. Yin, Y. Zhao, Z. Ren, Y. Sun, Q. Lei, J. Wang, L. Wang, T. Ji, P. Huai, W. Wen, X. Li, D. Zhu, R. Tai, *ACS Nano* **2022**, *16*, 12095.

[9] L. Liu, W. Yang, H. Chen, X. Chen, K. Zhang, Q. Zeng, S. Lei, J. Huang, S. Li, S. Peng, *Electrochim. Acta* **2022**, *410*, 140016.

[10] F. Niu, Z. Bai, Y. Mao, S. Zhang, H. Yan, X. Xu, J. Chen, N. Wang, *Chem. Eng. J.* **2023**, *453*, 139933.

[11] M. Hariram, M. Kumar, K. Awasthi, D. Sarkar, P. W. Menezes, *Dalton Trans.* **2023**, *52*, 12755.

[12] D. Xin, X. Zhang, Z. Zhang, J. Sun, Q. Li, X. He, R. Jiang, Z. Liu, Z. Lei, *Small* **2024**, *20*, 2403050.

[13] S. Qiao, W. Zhang, Y. Gao, X. Zhou, Q. Liang, Z. Xia, S. J. Yoo, J.-G. Kim, O. Bondarchuk, Z. Zhao, F. Liu, X. Ge, C. Huang, H. Yang, H. Pan, W. Zheng, *Acta Mater.* **2024**, *281*, 120370.

[14] M. Niu, W. Xin, L. Zhang, M. Yang, Y. Geng, X. Xiao, H. Zhang, Z. Zhu, *Inorg. Chem. Front.* **2024**, *11*, 2272.

[15] F. Niu, Z. Bai, J. Chen, Q. Gu, X. Wang, J. Wei, Y. Mao, S. X. Dou, N. Wang, *ACS Nano* **2024**, *18*, 6487.

[16] Q. Liu, J. He, J. Xie, H. Zhang, H. Wu, G. Wang, X. Lu, Z. Yang, *Nano Energy* **2024**, *127*, 109780.

[17] W. Yang, L. Mou, B. Xiao, J. Chen, D. Wang, S. Peng, J. Huang, *ACS Appl. Mater. Interfaces* **2023**, *15*, 51231.

[18] X. Wang, S. Zhang, R. Yang, S. Bai, J. Li, Y. Wu, B. Jin, X. Jin, M. Shao, B. Wang, *Nano Res.* **2023**.

[19] Z. Lv, Y. Tan, Y. Kang, J. Yang, X. Cheng, W. Meng, Y. Zhang, C. C. Li, J. Zhao, Y. Yang, *Sci. China Chem.* **2023**, *66*, 1537.
